# The Effectiveness of Computerized Cognitive Training in Patients With Poststroke Cognitive Impairment: Systematic Review and Meta-Analysis

**DOI:** 10.2196/73140

**Published:** 2025-06-12

**Authors:** Min Gao, Lu Huang, Jiang Yi, Tianqi Zhang, Guangyao Zhu, Qi Zhang, Jiaxiao Tian, Rongxuan Zhao, Xiaoqin Duan, Zhongliang Liu

**Affiliations:** 1 Department of Rehabilitation Medicine The Second Hospital of Jilin University Changchun, Jilin Province China; 2 School of Nursing Jilin University Changchun, Jilin Province China; 3 School of Automotive Engineering Jilin University Changchun China

**Keywords:** computerization, cognitive training, post-stroke cognitive impairment, cognitive function, meta-analysis.

## Abstract

**Background:**

Stroke often triggers poststroke cognitive impairment (PSCI) within 6 months, impairing memory, attention, and executive function while exacerbating physical disabilities and mortality. Computerized cognitive training (CCT) is a promising treatment approach. Compared to traditional methods, CCT provides cost-effective, easily accessible, personalized, and repetitive rehabilitation training. Therefore, we performed a systematic review of the efficacy of CCT to improve cognitive function in patients with PSCI and supplemented the findings with a meta-analysis.

**Objective:**

This study aimed to evaluate the efficacy of CCT in comparison to usual care or routine rehabilitation for patients with PSCI, with the aim of providing clinicians and therapists with more effective and convenient therapeutic options.

**Methods:**

A comprehensive systematic search was performed across multiple databases, including PubMed, Web of Science, Embase, Cochrane Library, and Scopus, to identify randomized controlled trials conducted between 2010 and June 2024 that used CCT to enhance cognitive function in patients with PSCI. The primary outcome of interest was cognitive function, evaluated using a standardized cognitive function scale, while secondary outcomes included assessments of patients’ activities of daily living and motor function. The risk of bias was evaluated using the Cochrane Risk of Bias tool, and the quality of evidence was appraised in accordance with the Grading of Recommendations, Assessment, Development, and Evaluations (GRADE) framework. Meta-analysis was conducted using RevMan 5.3 software published by The Cochrane Collaboration.

**Results:**

A total of 19 trials were included in the meta-analysis (n=875 participants). The findings provided moderate- to high-quality evidence indicating that CCT significantly enhances general cognitive function (15 trials, standardized mean difference [SMD]= 0.46, 95% CI 0.21-0.71; *P*<.001, *I*^2^=60%), attention (11 trials, SMD=–0.45, 95% CI –0.64 to –0.25, *P*<.001, *I*^2^=0%), executive function (6 trials, SMD=0.39, 95% CI 0.12-0.67; *P*=.01, *I*^2^=0%), and quality of life of PSCI patients (9 trials, SMD=0.34, 95% CI 0.15-0.53; *P*<.001, *I*^2^=6%). However, low- to very low–quality evidence indicated that CCT has limited improvement in memory, language function, and motor function in patients with PSCI.

**Conclusions:**

Based on moderate to severe levels of evidence, we conclude that CCT can significantly enhance general cognitive function, attention, executive function, and quality of life in PSCI patients. In addition, short-term high-frequency training was more effective than long-term low-frequency training. This review demonstrates CCT's effectiveness on cognitive function in patients with PSCI, though results vary due to differing intervention programs and CCT systems. Future research should increase sample sizes based on CCT system types and offer intensive, tailored training multiple times weekly. In addition, a structured maintenance plan is essential to sustain long-term benefits and prevent regression.

**Trial Registration:**

PROSPERO CRD42024573594; https://www.crd.york.ac.uk/PROSPERO/view/CRD42024573594

## Introduction

### Background

Stroke has emerged as the second leading cause of mortality and disability globally [1,2]. Poststroke cognitive impairment (PSCI) refers to a series of syndromes that meet the diagnostic criteria for cognitive impairment within 6 months following the clinical event of stroke [3,4]. The prevalence of cognitive deficits among stroke survivors ranges from 17.6% to 83%, with considerable variability across studies conducted in different countries. This variability is attributed to differences in stroke type, timing of assessments, study settings, population characteristics, and variations in cognitive tests and cutoff points [4,5]. PSCI is the most prevalent complication associated with stroke, leading to impairments in attention, executive function, memory, language, and visuospatial abilities (spatial awareness and visual processing). In addition, it exacerbates motor disorders, increases disability and mortality rates, hinders rehabilitation progress, and severely impacts the daily living abilities of patients [6]. If not effectively managed, PSCI can result in abnormal mental behaviors (eg, mood swings, apathy, anxiety, depression, hallucinations, and delusions) and may even progress to rapidly advancing dementia [3,4,7]. Consequently, PSCI poses significant challenges to both families and society at large.

The clinical management of PSCI primarily encompasses the intervention and prevention of established risk factors, pharmacological treatment, and cognitive training [4,8-11]. Notably, evidence-based management of risk factors for PSCI, alongside interdisciplinary research efforts focused on primary prevention, is critically needed [8,12]. Numerous studies have demonstrated that the risk factors for PSCI encompass a wide range of variables. These include patient-specific characteristics, such as age, gender, and genetic predispositions, as well as stroke-related factors, including the type of stroke, lesion location, and recurrence. Furthermore, vascular and metabolic conditions, such as hypertension, diabetes, hyperlipidemia, and atrial fibrillation, significantly contribute to the risk profile, alongside other determinants such as inflammation, oxidative stress, and psychological factors [5,13,14]. The pharmacological agents recommended for treatment are primarily those used for cognitive impairments associated with Alzheimer disease; however, their efficacy and safety in the context of PSCI remain unverified [15,16]. Traditional cognitive training has been proven to be effective in most studies. Cognitive training aims to promote neural plasticity and functional reorganization by systematically and repeatedly stimulating the neural network related to attention, memory, and executive function in the brain [17]. Nonetheless, challenges persist, including the time-consuming and labor-intensive nature of these interventions, as well as suboptimal patient compliance among individuals with cognitive impairments [8].

In recent years, with the rapid development of computer technology, computerized cognitive training (CCT) has gradually replaced traditional cognitive training. CCT can simultaneously mobilize multiple senses of patients through multimedia stimulation channels (such as visual puzzles, auditory memory games, and interactive simulation) and information resources [18]. The rationale for CCT is based on cognitive psychology and neuroscience research and focuses on the activation of brain plasticity through repetitive practice and feedback mechanisms. Compared with “pencil-and-paper” cognition training tasks, CCT can enhance the immersion and interactivity of the cognitive training process, thus promoting the consolidation of the training effect [19]. At the same time, CCT can design different tasks according to different cognitive domains. In the study of Withiel et al [20], games targeting memory function were applied to improve the cognitive domains of attention function. In addition, personalized parametric training tasks can be used to improve cognitive domains such as attention and executive function [21]. Furthermore, the low implementation cost, high availability, and accessibility of CCT have contributed to its increasing adoption both domestically and internationally.

In this literature, CCT is frequently used for patients with mild cognitive impairment, dementia, and Parkinson disease, demonstrating varying levels of enhancement across different cognitive domains [22-25]. A meta-analysis conducted by Fernández López and Antolí [26] revealed that CCT significantly improved visual and verbal working memory in individuals with acquired brain injury, although it did not enhance attention or executive function. Another meta-analysis indicates that higher-intensity CCT, including remote unsupervised training, is advantageous for general cognitive function in patients with Parkinson disease [25]. Research by Hill et al [22] found CCT to be effective in enhancing general cognition, memory, and attention in older patients with mild cognitive impairment, as well as in improving psychosocial functioning, including alleviation of depressive symptoms, albeit with limited impact on executive function. In addition, there is insufficient evidence to support the efficacy of CCT in enhancing cognitive function in patients with dementia. Although more and more studies have investigated the beneficial impact of CCT on cognitive function, there is still a lack of high-quality evidence on cognitive function and different cognitive domains in patients with PSCI.

### Aims of This Review

The objective of this review is to systematically evaluate existing clinical studies and conduct a meta-analysis to compare the effectiveness of CCT with alternative interventions, such as usual care and routine rehabilitation training, for patients experiencing poststroke cognitive impairment. At the same time, we hope to provide new ideas and new methods for clinicians and therapists.

## Methods

### Study Registration

This review was conducted and reported based on the recommendations of the PRISMA (Preferred Reporting Items for Systematic Reviews and Meta-analyses; see checklist in Multimedia Appendix 1) [27]. The systematic review with meta-analysis had been registered in the PROSPERO database (CRD42024573594).

### Search Strategy

We searched PubMed, Web of Science, Embase, Cochrane Library, and Scopus databases from 2010 to June 2024. The complete search strategies, developed with the assistance of the research librarian, are presented in the Multimedia Appendix 2. All publications in English were searched. Informed by preliminary scoping of the literature, our keywords and MeSH (Medical Subject Headings) terms related to “stroke,” “Cognitive Dysfunction,” “training,” “compute*” and “randomized controlled trial.” In addition, keywords and subject words are combined using Boolean operators. Reference lists of selected articles were independently screened to detect additional studies not included in the original search.

### Selection Criteria

Screen titles, abstracts, and methods using inclusivity and exclusivity criteria as PICOS (Population, Intervention, Comparison, and Outcome). The inclusion criteria and exclusion criteria for this study were as mentioned in Textbox 1.

**The inclusion criteria and exclusion criteria.**

**Inclusion Criteria**

- The study must be a published randomized controlled trial (RCT);
- the study population must be poststroke cognitive impairment (PSCI), defined as cognitive dysfunction resulting from a stroke;
- the intervention group must have received computerized cognitive training (CCT);
- the control group must have received interventions other than CCT, such as conventional treatment, traditional paper-and-pencil–based cognitive training, or health education;
- the study must report at least one cognitive assessment (general cognitive function, attention, memory, executive function, language, and other cognitive domains) was included, and other indicators could or could not include quality of life and limb motor function.

**Exclusion criteria**

- patients whose baseline conditions differed significantly within the study;
- studies that did not report original outcome metrics or where relevant outcome metrics could not be obtained despite contacting the authors;
- articles in which the study population included patients with other cognitive disorders, such as Alzheimer disease;
- studies where the intervention group received additional interventions alongside CCT, such as CCT combined with medication or acupuncture, compared to the control group treatment;
- animal experimental studies, case reports, or protocols;
- articles that were either repeatedly published or retracted;
- studies for which only abstracts were available and the full text could not be retrieved;
- publications not in English.

### Data Extraction

The literature retrieved was screened by 2 researchers in accordance with the inclusion and exclusion criteria. Baseline characteristics and outcome indicators were extracted using standardized forms. In cases of differing opinions, resolution was achieved through discussion or consultation with a third researcher. The descriptive data extracted from each trial included baseline characteristics (such as authors, year of publication, and country), participant characteristics (including intervention population and sample size), intervention characteristics (such as setting, frequency, duration, and any additional components like computer systems), and outcome indicators. For outcome indicators, the mean and SD were collected for each group. If studies did not report the relevant data, the corresponding authors were contacted to obtain any missing data.

### Data Analysis and Synthesis

The primary outcome measure was the assessment of cognitive function, while the secondary outcome measures included evaluations of patients' activities of daily living and motor function. In instances where multiple scales were used to assess the same outcome measure, we prioritized those indicators with higher reliability or sensitivity. Detailed prioritization criteria are provided in Multimedia Appendix 3. For certain scales, lower scores indicated better functioning, for example, Trail Making Test-A and Shulte's table; thus, we applied a negative effect size to this indicator to denote improved functioning in the trial group compared to the control group. Post–follow-up assessments were also extracted for studies that reported follow-up.

Statistical heterogeneity tests and comprehensive data analyses were conducted using Review Manager version 5.3, following the Cochrane guidelines. Standardized mean differences (SMDs) and 95% CIs were calculated for continuous data due to the use of different scales across the included trials for the same outcome. Given the substantial heterogeneity in the intervention characteristics of the studies, a random-effects model was used to derive pooled estimates for all outcome measures. Differences were deemed statistically significant when *P*<.05.

### Subgroup Analysis

Subgroup analyses were performed to examine the effect of intervention duration (>6 weeks and ≤6 weeks) on cognitive function and cognitive domains.

### Risk-of-Bias Assessment

We evaluated the risk of bias in trials using the Cochrane Risk of Bias tool (version 2) as outlined in the Cochrane Handbook, focusing on the following domains: the randomization process, deviations from intended interventions, missing outcome data, outcome measurement, and selective reporting of outcomes [28]. Each domain was classified as having a high, low, or unclear risk of bias. An overall risk of bias assessment was subsequently determined based on the individual domain assessments, typically reflecting the highest level of bias identified in any domain. In instances of disagreement or discrepancy during the quality assessment process, a third researcher was tasked with verification and resolution.

### Certainty Assessment

To evaluate the quality of evidence, we used the Grading of Recommendations, Assessment, Development, and Evaluations (GRADEpro GDT) methodology [29]. In total, 2 independent assessors used the GRADEpro GDT software to appraise the quality of evidence, categorizing it as very low, low, moderate, or high. Footnotes were provided to elucidate the rationale for any downgrading of evidence, which included considerations of risk of bias, inconsistency, indirectness, imprecision, and publication bias.

### Additional Analyses

To further investigate the robustness of the results and identify potential sources of heterogeneity, we performed several additional post hoc analyses. A sensitivity analysis was conducted to evaluate the stability of the meta-analysis outcomes by sequentially excluding individual studies to assess their influence on the overall results. For meta-analyses comprising a sufficient number of studies (n≥10), we intended to evaluate publication bias through the use of funnel plots and Begg tests [30]. *P*<.05 was considered statistically significant.

## Results

### Search Outcomes

**Figure 1:**
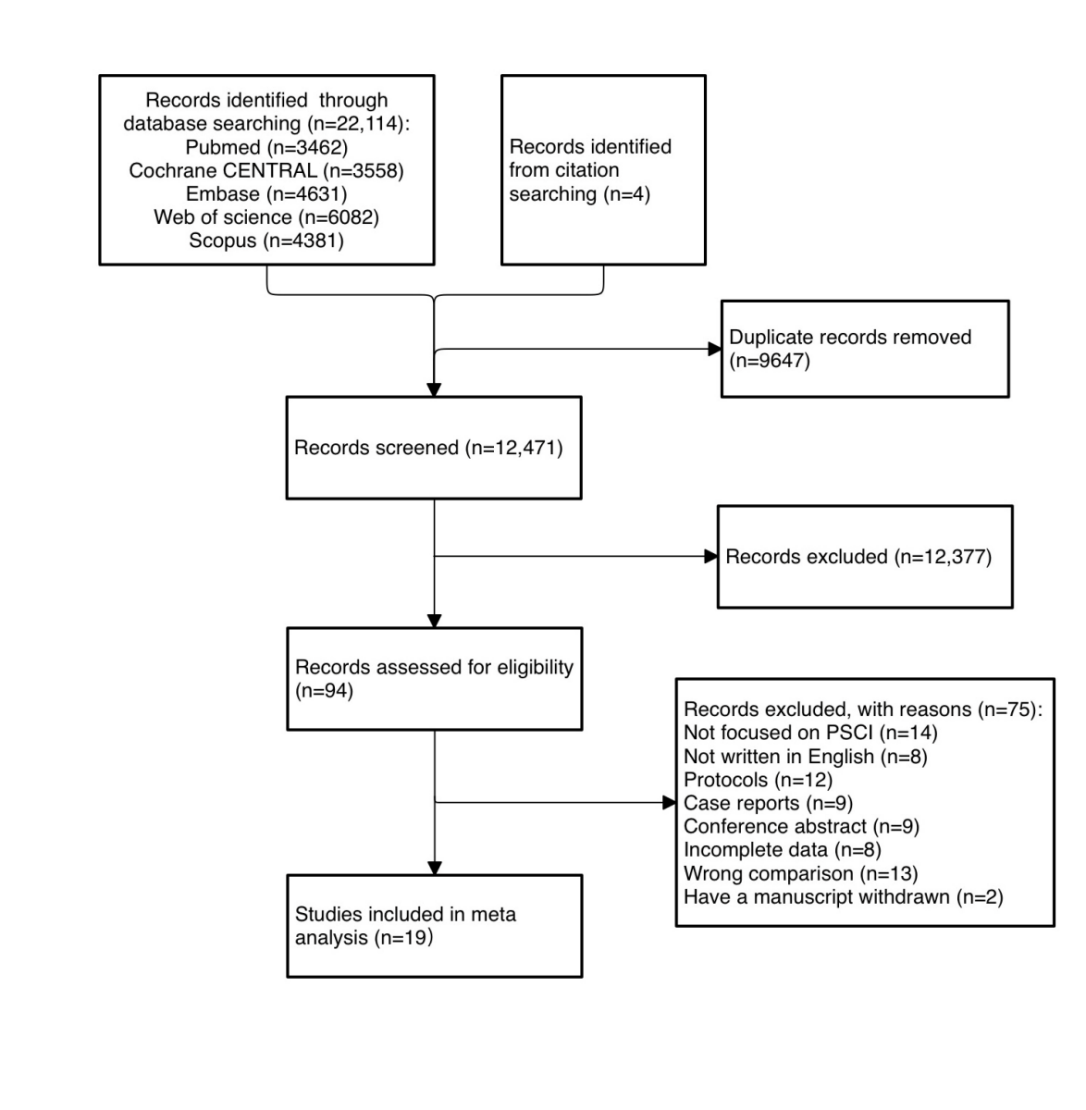
PRISMA (Preferred Reporting Items for Systematic Reviews and Meta-analyses) flow diagram. PSCI: poststroke cognitive impairment.

The search results are presented in Figure 1. An initial retrieval yielded 22,114 studies from 5 electronic databases. Of these, 9647 studies were excluded due to duplication. A meticulous screening of the titles and abstracts of the remaining 12,471 studies resulted in the exclusion of 12,377 studies that did not satisfy the research criteria. Consequently, 94 studies underwent a comprehensive review. Ultimately, 19 randomized controlled trials (RCTs) were included in the meta-analysis.

### Characteristics of Included Studies

This review incorporated a total of 19 RCTs encompassing 875 participants. The participants' ages ranged from 27 to 84 years. Regarding the duration and frequency of the intervention, 9 of the trials had a duration of less than or equal to 6 weeks [20,21,31-37], and 10 trials had a duration of more than 6 weeks [38-47]. Regarding the software used in CCT, 3 studies used Rehacom [32,38,40], 2 studies used Reh@City [21,34], 2 studies used Lumosity software [20,42], 2 studies used the Forbrain System [39,45]. The remaining 5 studies used various software, including NeuroActive [47], Erica software [46], Netblue Co, Ltd, Korea [35], the Rehabilitation Gaming System [31], and the Kinect System [43]. In total, 5 studies either did not specify the software used or used individually customized computer-assisted training programs [33,36,37,41,44]. Participants in the control groups primarily received conventional drug therapy alongside rehabilitation training, such as occupational therapy or traditional paper-and-pencil cognitive training. In terms of follow-up, 3 studies conducted assessments 1 month post intervention [21,42,47], 2 studies conducted assessments 3 months post intervention [31,43], and 1 study conducted assessments 1.5 months post intervention [20]. In total, 5 trials were supervised at home [20,39,43,45,47], and the rest were supervised in the hospital. Further details on the specific characteristics of the included studies are provided in Multimedia Appendix 4.

### Risk of Bias Assessment

**Figure 2:**
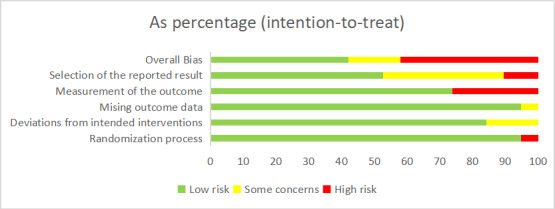
Risk of bias for all included studies.

**Figure 3:**
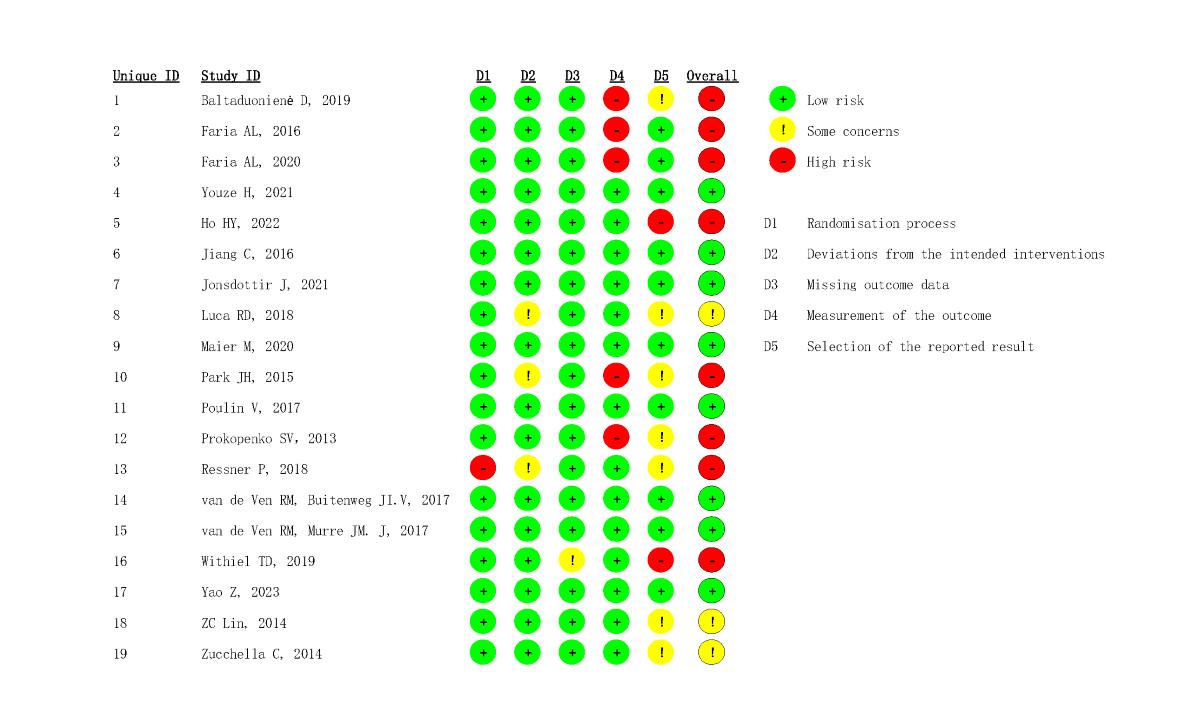
Summary of distribution of different biases. Green symbols indicate low risk of bias, yellow symbols indicate unclear risk of bias, and red symbols indicate high risk of bias.

Among the 19 studies, 3 trials exhibited some risk of bias [33,38,46], 8 trials were assessed as having a low risk of bias [31,36,39-41,43,45,47], and 8 trials were assessed as having a high risk of bias [20,21,32,34,35,37,42,44]. The reasons for the identified risk of bias included the lack of assessor blinding in 5 trials [21,32,34,35,37], the presence of randomization bias in one trial [44], and the absence of available protocols or complete outcome data reporting in 2 trials [20,42] (Figures 2 and 3 [20,21,31-47]).

### Intervention Effect

The GRADEpro GDT evidence level for the meta-analysis results indicated that the quality of evidence for attention and quality of life was high, the quality of evidence for executive function was medium, and the quality of evidence for other outcomes was low or very low. The primary factors contributing to the reduction in evidence quality were the risk of bias and inconsistency. A comprehensive GRADEpro GDT summary of the results is available in Multimedia Appendix 5.

### Meta-Analysis Results

#### Primary Outcome: General Cognitive

**Figure 4:**
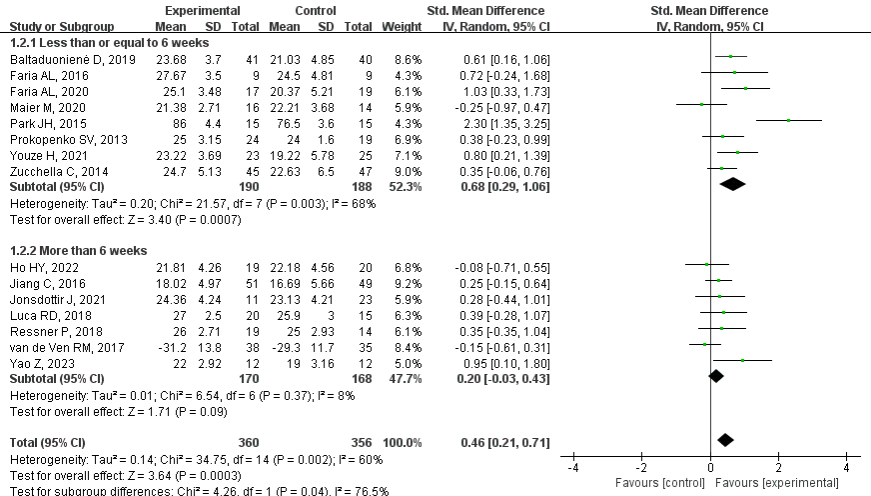
Effects of CCT on general cognitive function in patients with PSCI. CCT: computerized cognitive training, PSCI: poststroke cognitive impairment.

Based on moderate-quality evidence, a meta-analysis of 15 trials indicated that general cognitive function scores were superior in the CCT group than in the control group (SMD=0.46, 95% CI 0.21-0.71, *P*<.001, *I*^2^=60%). After excluding one study by Park and Park [35], the heterogeneity was reduced (*I*^2^=32%), while the pooled SMD remained largely unchanged (SMD=0.36, 95% CI 0.17-0.55; *P*<.001) (Multimedia Appendix 6). Upon analyzing the subgroups based on the intervention duration, it was observed that the CCT group exhibited improvements in general cognitive function when the intervention time was less than or equal to 6 weeks (SMD=0.68, 95% CI 0.29-1.06, *P*<.001, *I*^2^=68%). Conversely, there were no significant differences between groups when the intervention was extended more than 6 weeks (SMD=0.20, 95% CI –0.03 to 0.43, *P*=.09, *I*^2^=8%; Figure 4 [21,31-37,40-46]).

In the long term, a meta-analysis of 4 trials demonstrated no between-group difference in general cognitive (SMD=0.20, 95% CI –0.18 to 0.58, *P*=.3, *I*^2^=18%; Multimedia Appendix 7).

To enhance the evaluation of the results, subgroup analyses of general cognitive function were conducted based on intervention frequency, the intervention modality within the control group, and the modality of supervision for CCT training. The results indicated that short-term high-frequency and short-term low-frequency training were more effective than long-term high-frequency and long-term low-frequency training. The findings revealed no significant differences in the subgroup analysis, with the exception of the supervision modality (Multimedia Appendix 8).

#### Primary Outcome: Attention

**Figure 5:**
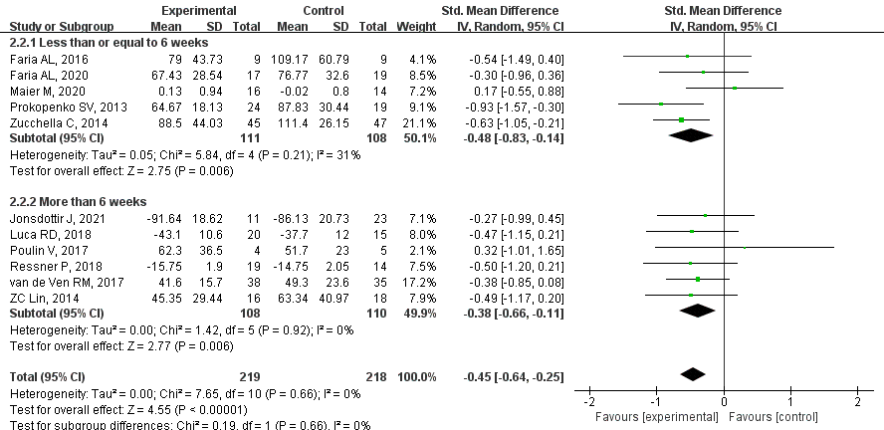
Effects of CCT on attention in patients with PSCI. CCT: computerized cognitive training, PSCI: poststroke cognitive impairment.

Based on high-quality evidence, a meta-analysis of 11 studies showed that the attention function of PSCI patients in the CCT group was significantly improved (SMD=–0.45, 95% CI –0.64 to –0.25, *P*<.001, *I*^2^=0%). Subgroup analysis showed that the CCT group had improved attention when the intervention time was less than or equal to 6 weeks (SMD=–0.48, 95% CI –0.83 to –0.14, *P*=.006, *I*^2^=31%). Attention was also improved at more than 6 weeks (SMD=–0.38, 95% CI –0.66 to –0.11, *P*=.006, *I*^2^=0%; Figure 5 [21,31,33,34,37-39,43,44,46,47]).

In the long term, a meta-analysis of 4 trials demonstrated no between-group difference in attention (SMD=–0.23, 95% CI –0.62 to 0.15, *P*=.23, *I*^2^=0%; Multimedia Appendix 7).

#### Primary Outcome: Memory

**Figure 6:**
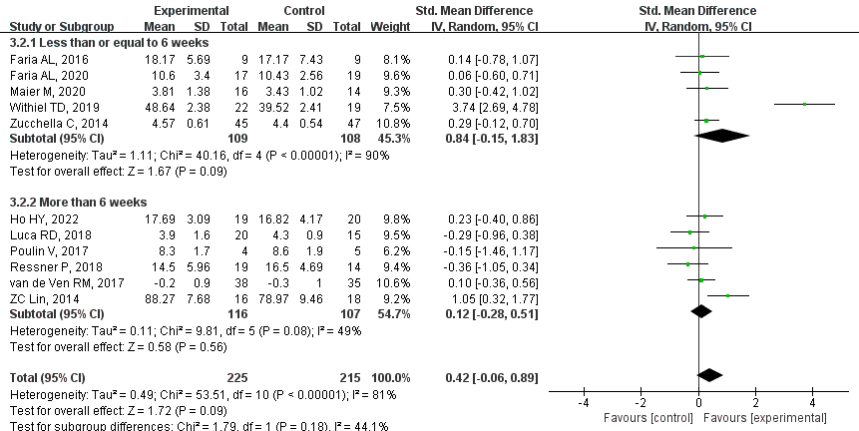
Effects of CCT on memory in patients with PSCI. CCT: computerized cognitive training; PSCI: poststroke cognitive impairment.

Based on very low-quality evidence, a meta-analysis of 11 studies showed no statistical difference in memory function between groups (SMD= 0.42, 95% CI –0.06 to 0.89, *P*=.09, *I*^2^=81%). After excluding one study by Withiel et al [20], the heterogeneity was reduced (I^2^=15%), and the pooled SMD changed significantly but were still not statistically significant (SMD=0.16, 95% CI –0.06 to 0.38; *P*=.15; Multimedia Appendix 6). Subgroup analysis showed that there was no significant difference between the groups when the intervention time was less than or equal to 6 weeks (SMD=0.84, 95% CI –0.15 to 1.83, *P*=.09, *I*^2^=90%). There were also no significant differences between groups at more than 6 weeks (SMD=0.12, 95% CI –0.28 to 0.51, *P*=.56, *I*^2^=49%; Figure 6 [20,21,31,33,34,38,39,42,44,46,47]).

In the long term, a meta-analysis of 5 trials demonstrated no between-group difference in memory (SMD=0.63, 95% CI –0.15 to 1.42, *P*=.11, *I*^2^=80%; Multimedia Appendix 7).

#### Primary Outcome: Executive Function

**Figure 7:**
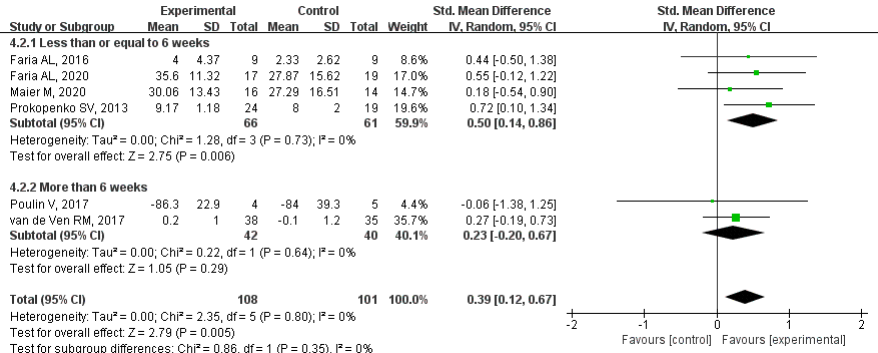
Effects of CCT on executive function in patients with PSCI. CCT: computerized cognitive training; PSCI: poststroke cognitive impairment.

Based on moderate quality evidence, a meta-analysis of 6 studies showed that executive function was significantly improved in patients with PSCI in the CCT group (SMD=0.39, 95% CI 0.12-0.67, *P*=.005, *I*^2=^0%). Subgroup analysis showed that the CCT group had improved executive function when the intervention duration was less than or equal to 6 weeks (SMD=0.50, 95% CI 0.14-0.86, *P*=.006, *I*^2^=0%). At more than 6 weeks, there was no significant difference between the groups (SMD=0.23, 95% CI –0.20 to 0.67, *P*=.29, I^2^=0%; Figure 7 [21,31,34,37,39,47]).

In the long term, a meta-analysis of 3 trials demonstrated no between-group difference in executive function (SMD=0.29, 95% CI –0.17 to 0.75, *P*=.21, *I*^2^=0%; Multimedia Appendix 7).

#### Primary Outcome: Language

**Figure 8:**
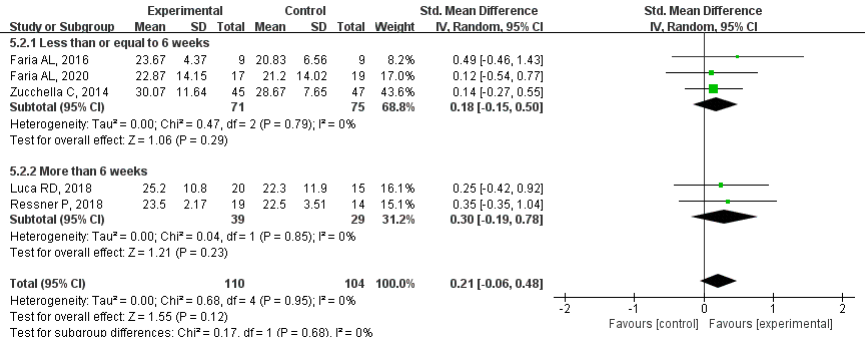
Effects of CCT on language in patients with PSCI. CCT: computerized cognitive training; PSCI: poststroke cognitive impairment.

Based on low-quality evidence, a meta-analysis of 5 studies showed no statistical difference in language function between groups (SMD=0.21, 95% CI –0.06 to 0.48, *P*=.12, *I*^2^=0%). Subgroup analysis showed that there was no significant difference between the 2 groups when the intervention time was less than or equal to 6 weeks (SMD=0.18, 95% CI –0.15 to 0.50, *P*=.29, *I*^2^=0%). There was also no significant difference between the groups when the study was more than 6 weeks (SMD=0.30, 95% CI –0.19 to 0.78, *P*=.23, *I*^2^=0%; Figure 8 [21,33,34,44,46]). Because of the small number of follow-up studies, a long-term analysis was not performed.

#### Secondary Outcome: Quality of Life

**Figure 9:**
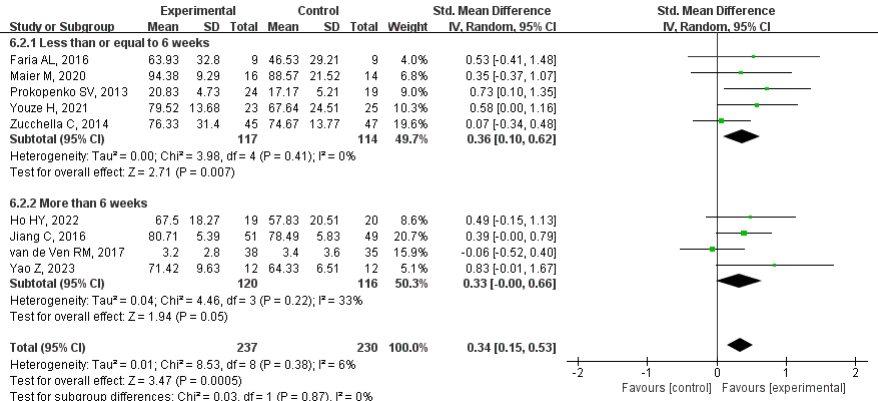
Effects of CCT on quality of life in patients with PSCI. CCT: computerized cognitive training; PSCI: poststroke cognitive impairment.

Based on high-quality evidence, the meta-analysis of 9 studies showed that the quality of life of PSCI patients in the CCT group was significantly improved (SMD=0.34, 95% CI 0.15-0.53, *P*<.001, *I*^2^=6%). Subgroup analysis was performed according to the intervention duration, and the CCT group showed improvement in quality of life when the intervention time was less than or equal to 6 weeks (SMD=0.36, 95% CI 0.10-0.62, *P*=.007, *I*^2^=0%). At more than 6 weeks, there was no significant difference between the groups (SMD=0.33, 95% CI –0.00 to 0.66, *P*=.05, *I*^2^=33%; Figure 9 [31,33,34,36,37,40-42,45]). Because of the small number of follow-up studies, a long-term analysis was not performed.

#### Secondary Outcome: Motor Function

**Figure 10:**
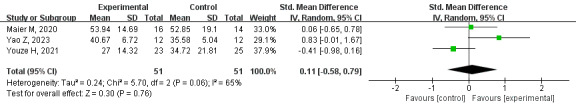
Effects of CCT on motor function in patients with PSCI. CCT: computerized cognitive training; PSCI: poststroke cognitive impairment.

Based on low-quality evidence, a meta-analysis of 3 trials showed no statistical difference in motor function between CCT and control groups (SMD=0.11, 95% CI –0.58 to 0.79, *P*=.76, *I*^2^=65%; Figure 10 [31,36,41]). Subgroup analyses and long-term analyses were not performed due to the small number of studies.

### Additional Analyses

Publication bias was evaluated for general cognitive function, attention, and memory across more than 10 included studies, as detailed in Multimedia Appendix 9. A visual inspection of the funnel plots indicated no significant asymmetry. Furthermore, the Begg test results demonstrated the absence of publication bias for general cognitive function (*P*=.09), attention (*P*=.28), and memory (*P*=.53), with all *P*>.05.

## Discussion

### Principal Findings

This review synthesizes the evidence concerning the impact of CCT on general cognitive function, specific cognitive domains, daily activities, and motor function in individuals with PSCI. Despite variations in the CCT systems used, based on moderate to severe levels of evidence, the findings indicate that CCT is generally effective in enhancing general cognitive function, particularly in the domains of attention and executive function, as well as in improving the quality of life for patients with PSCI.

### Mechanisms by Which CCT Improves Cognitive Function

Studies have shown that CCT can significantly enhance the recovery of cognitive function in PSCI through the combined influence of neuroplasticity, neurofeedback, task design, and other aspects. Vermeir et al [48] demonstrated that CCT facilitates the reorganization of brain function by incorporating challenging cognitive tasks that activate adjacent or contralateral brain regions relative to the stroke-affected area. Concurrently, CCT systems typically provide immediate feedback, enabling patients to assess their task performance in real time and adjust their strategies accordingly. Yuan et al [49] highlighted the critical role of positive feedback and reward mechanisms in bolstering patient engagement and motivation for training. Moreover, CCT tasks frequently involve the stimulation of multiple sensory modalities, including visual, auditory, and tactile inputs. This cross-modal training approach engages multiple brain regions concurrently, thereby enhancing the cognitive function of patients.

Neuroimaging investigations have significantly advanced our understanding of neuroplasticity induced by CCT in individuals who have experienced a stroke. For instance, Lin et al [38] used resting-state functional magnetic resonance imaging to examine the impact of CCT on patients with poststroke cognitive impairment. Their findings indicated an enhancement in functional connectivity between the hippocampus and the frontal and parietal regions following a 10-week training regimen. Furthermore, a longitudinal study conducted by Nyberg et al [50] explored the association between white matter microstructure and cognitive performance in patients with stroke undergoing working memory training. Although no significant alterations in white matter integrity were observed post training, the study underscores the intricate nature of neural adaptation and underscores the necessity for further research to elucidate the structural correlates of cognitive enhancement.

### Principal Results

In this review, we found that there was a large heterogeneity in the meta-analysis of general cognitive function (*I*^2^=73%). Upon excluding the study by Park and Park [35], the heterogeneity reduced moderately (*I*^2^=32%), although the effect size was reduced to 0.36. This reduction in effect size may be attributed to the more pronounced improvement in general cognitive function observed in the study by Park and Park [35] compared to others. This discrepancy could be due to variations in the scales used to measure functional outcomes. Specifically, the study by Park and Park [35] used the Loewenstein Occupational Therapy Cognitive Assessment (LOTCA) scale to assess patients' general cognitive function. Unlike the Montreal Cognitive Assessment and the Mini-Mental State Examination, the LOTCA scale evaluates not only cognitive function but also perceptual and daily functional abilities, involving more complex cognitive domains, particularly visuospatial cognition. The extended evaluation time required by the LOTCA scale allows for a more comprehensive analysis, potentially offering a better reflection of functional improvements in patients with PSCI [51,52].

CCT improves the function of multiple cognitive domains in patients with PSCI by employing targeted cognitive tasks designed to stimulate neuroplasticity. The mechanisms underlying improvements are intricately linked to the physiological processes of the brain, including neural remodeling, functional compensation, and cognitive stimulation. Consistent with previous research [53-55], our review found significant benefits of CCT in improving attention and executive function in PSCI patients. The regulation of attention and executive functions primarily relies on the prefrontal cortex, parietal lobe, and the network of connections between them [56,57]. These regions are involved in key processes such as decision-making, planning, task-switching, and information filtering. It promotes long-term potentiation and synaptic plasticity through repeated practice to achieve neural network reorganization and functional recovery [58]. Neuroimaging studies have shown that dense CCTS can improve brain functional connectivity, especially network synergies between the prefrontal cortex and other cognitive regions. Immediate feedback and reward mechanisms also contribute to enhanced self-monitoring and adaptation in the prefrontal cortex [59]. In addition, high-frequency CCT can enhance the neuroplasticity of key brain regions, for example, by upregulating the expression of brain-derived neurotrophic factor, which promotes the formation and strengthening of synaptic connections [60]. This mechanism is particularly evident in the prefrontal and parietal networks, which in turn improve attention and executive function [61,62].

Memory functions mainly rely on the hippocampus and its associated medial temporal structures, whereas language functions mainly involve the superior temporal gyrus, Broca's area, and Wernicke's area in the left hemisphere. These regions are structurally complex and the neural circuits are difficult to rapidly modulate. Compared with prefrontal networks, these regions may require longer duration or more task-specific design to activate effective neural plasticity in the face of the same training, and thus have limited effect on the improvement of memory and language function [63,64]. Conventional task designs of CCTS may fail to achieve adequate levels of stimulation, and these areas may require specifically designed memory or language training tasks or in combination with assistive techniques such as noninvasive brain stimulation to further activate their neuroplasticity [65]. In addition, incorporating multimedia stimuli such as visual puzzles, auditory memory games, or interactive simulations may provide more dimensional stimuli to activate the hippocampus and language areas, thereby potentially improving training outcomes in these areas [49,66].

A meta-analysis conducted by Kazinczi et al [65] demonstrated that CCT exerts a significant impact on memory function in stroke patients, as assessed by the Digit Span Backward Test. Conversely, our findings indicate that CCT has a limited effect on improving memory and language functions in patients with PSCI. This may be due to the fact that the Digit Span Backward Test is a widely used measure of working memory, requiring participants to recall a sequence of digits in reverse order. This task necessitates the retention and manipulation of information, engaging both central executive functions and short-term memory capacity. Our study extends beyond the digit span test by incorporating assessments such as Addenbrooke's Cognitive Examination memory subscale and Goal Attainment Scaling, which evaluate memory function through various high-level cognitive behaviors, including directed attention and complex decision-making. The observed discrepancies in findings may be attributable to differences in neural mechanisms and functional brain networks. Notably, upon excluding the study by Withiel et al [20], heterogeneity was reduced (*I*^2^=15%), and the effect size showed a notable change (SMD=0.16), indicating an improvement in memory function, although this change was not statistically significant. The Goal Attainment Scaling used in this study is primarily employed for goal setting and outcome evaluation in rehabilitation interventions, demonstrating robust reliability and validity. Nevertheless, its subjective and individualized nature limits its direct validity in assessing specific cognitive domains, such as memory or attention, which accounts for its infrequent application in detailed cognitive function assessments. Similar to our results, previous studies have indicated that CCT may facilitate improvements in working memory and short-term information retention; yet, it appears to be less effective in enhancing long-term and contextual memory [42,67,68].

In addition, our results showed that CCT could enhance the quality of life in patients with PSCI. Cognitive impairment after stroke can substantially diminish patients' quality of life, affecting their self-care capabilities, emotional well-being, and social interactions [15]. By improving cognitive function and emotional state, CCT can indirectly contribute to an improved quality of life. The immediate feedback and reward mechanisms inherent in CCT training facilitate patients' perception of progress and achievement, thereby boosting self-confidence and fostering positive emotions, which are essential for improving overall quality of life [54,62,69].

In clinical practice, cognitive impairment and motor dysfunction frequently coexist in patients who have experienced a stroke. Research indicates a correlation between motor functions, such as grip strength disorders, gait, and balance, and general cognitive impairment, including executive dysfunction and memory impairment [70-74]. This review suggests that while CCT may not directly enhance motor function, cognitive abilities are crucial for limb and motor rehabilitation. Cognitive impairments, such as memory deficits or executive dysfunction, can influence the progression of motor rehabilitation [15,70,75,76]. Consequently, these factors should be considered when developing targeted interventions for the stroke population. Other studies have shown that aerobic exercise can enhance the activation of the left dorsolateral prefrontal cortex, thereby improving executive function [77-79]. Thus, integrating aerobic exercise with CCT in clinical practice may yield beneficial therapeutic effects on the recovery of both motor and cognitive functions in patients with PSCI.

### Discussion of Subgroup Analyses

In subgroup analyses according to intervention duration, we found that the short-term intervention (≤6 weeks) was significantly better than the long-term intervention (> 6 weeks) with respect to global cognitive function, attention, executive function, and quality of life.

Due to the insufficient number of articles, we performed additional group analyses only for overall cognitive function, taking into account the frequency and duration of CCT interventions. Specifically, we divided the study into four different groups: (1) high frequency and short duration (>3 sessions per week, ≤6 weeks), (2) high frequency and long duration (>3 sessions per week, >6 weeks), (3) low frequency and short duration (≤3 sessions per week, ≤6 weeks), and (4) low frequency and long duration (≤3 sessions per week, >6 weeks)

Additional subgroup analyses indicated that short-term high-frequency and short-term low-frequency training was more effective than long-term high-frequency and long-term low-frequency training. However, it is important to note that the evidence for short-term low-frequency training is limited, as it is based on only 2 studies, which may constrain the generalizability of these findings.

By analyzing the characteristics of the included studies, we found that the frequency of weekly interventions was usually 5 or more days for studies that lasted short, while the frequency of weekly interventions was lower, 2-5 days, for studies that lasted long. The results were consistent with a previous study [80]. This may be because short-term high-frequency training can reduce interference and forgetting, thus making newly acquired skills and cognitive strategies more stable. In addition, training of too long duration may lead to poor patient compliance and difficulty in maintaining consistent effort and attention, thereby reducing the overall intervention effect [81-83]. In terms of neural plasticity, high-frequency training can activate and strengthen the connections between neural networks more rapidly, promote synaptic plasticity and long-term potentiation, and thus reorganize the function of the damaged area more quickly. This “mass intensive” training can rapidly trigger the release of nerve growth factors such as brain-derived neurotrophic factor, further accelerating the cognitive repair process in the brain [80,82,84]. Therefore, short-term high-frequency cognitive training can be considered in clinical practice to achieve more rapid and significant cognitive function improvement. For instance, protocols involving 30-minute sessions conducted five times per week over five weeks have demonstrated significant enhancements in working memory and processing speed. However, it is essential to tailor the intensity and duration of CCT to individual patient capacities to avoid cognitive fatigue. Adjustments should be made based on the patient's baseline cognitive function, endurance, and overall health status.

We conducted subgroup analyses comparing active control groups (eg, traditional cognitive training) with passive control groups (eg, usual care or waitlist). The results indicate that although both types of control groups demonstrated significant improvements in general cognitive function, the effect sizes were marginally larger in studies using passive controls. This observation is consistent with existing literature, which suggests that passive control conditions may inadequately account for placebo effects or participant expectations, potentially resulting in inflated effect sizes [85]. In contrast, active controls, by offering an alternative form of cognitive engagement, may provide a more rigorous comparison, thereby producing more conservative estimates of intervention efficacy. It is proposed that CCT be integrated with conventional cognitive training and other rehabilitation modalities in clinical settings to enhance the overall therapeutic outcomes. Empirical evidence supports that the amalgamation of CCT with traditional interventions, such as paper-and-pencil exercises, physical therapy, and occupational therapy, significantly enhances various cognitive domains, including attention, memory, and executive function [54]. Moreover, incorporating CCT into a multidisciplinary rehabilitation program aligns with the recommendations outlined in pertinent guidelines for comprehensive treatment, which involve collaboration among physical therapists, occupational therapists, speech and language therapists, and clinical psychologists.

Furthermore, the training effect under hospital supervision was significantly superior to that under home supervision. It is recommended that CCT for cognitive function enhancement be conducted under professional supervision whenever possible.

Due to the diverse range of CCT software used and the limited number of studies using each specific software, subgroup analyses regarding their effects were not conducted. Among the more frequently used programs, RehaCom, Reh@City, and Lumosity have demonstrated some efficacy in enhancing specific cognitive functions in patients with stroke, albeit through differing methodologies. RehaCom offers structured, domain-specific exercises; Reh@City provides immersive, ecologically valid simulations of everyday activities; and Lumosity features a variety of games targeting different cognitive skills. However, there is insufficient evidence supporting Lumosity's effectiveness in cognitive rehabilitation poststroke [21,86]. Clinicians are advised to select CCT based on the individual needs of patients.

### Long-term Follow-up Analysis

Our study did not reveal any between-group differences in general cognition or specific cognitive domains at follow-up intervals ranging from 1 to 3 months. Previous research indicates that while cognitive performance may improve during the first year poststroke, there is a propensity for cognitive decline in the subsequent years. A meta-analysis conducted by the Stroke and Cognition Consortium identified significant declines in overall cognition between 1 and 3 years post stroke, with notable deterioration in memory and attention [87]. Similarly, the ARCOS-IV study reported that 84% of stroke survivors experienced cognitive impairment, including poststroke dementia, at a 4-year follow-up [88]. In light of these findings, we recommend that future studies incorporate follow-up assessments at multiple intervals, such as 1, 3, and 6 months, as well as, 1, 3, and 5 years post intervention. This approach would facilitate a more comprehensive evaluation of the long-term sustainability of cognitive improvements. In addition, it could inform the development of maintenance strategies or reinforcement courses to preserve cognitive gains over the long term.

### Quality of the Evidence

In this study, we used the Cochrane Risk of Bias tool to rigorously assess the methodological quality of the included studies, and GRADEpro GDT was used to grade the quality of evidence for each outcome. It should be noted that the quality of evidence is directly related to our confidence in the reliability of the study's conclusions. For example, a rigorously randomized study with a low risk of bias and good consistency of results (GRADE rated as high quality or moderate-high quality evidence) could make a stronger clinical recommendation for the effect of a CCT on improving attention, memory, and executive functioning. However, caution should be exercised in formulating clinical recommendations for studies with a high risk of bias, imprecision, or inconsistent results (GRADE, low-quality evidence), and recommendations should be made only as supplementary or conditional. In order to ensure the scientific and safety of clinical practice, medical staff should comprehensively consider the quality of all the evidence, formulate personalized plans based on the individual situation of patients, and closely monitor the intervention effect when necessary, to adjust the rehabilitation strategy in time.

### Limitations

Our systematic review and meta-analysis are subject to several limitations. First, there is variability in the diagnostic criteria for cognitive impairment among the studies included in this review, which may introduce a certain degree of heterogeneity. Second, there is considerable variation in the frequency and intensity of CCT interventions, including differences in the duration of each training session and the overall intervention period, potentially affecting the reliability of the results. Third, there is a scarcity of studies with long-term follow-up outcomes, with only 6 trials providing follow-up data extending from one to 3 months. Finally, the selection was limited to English publications, ignoring those in other languages, which might lead to an underestimation of the worth of non-English publications.

### Future Research

Based on the results of the meta-analysis, we propose that future research should establish standardized assessment criteria for each cognitive domain and identify the most effective rating scales to enhance comparability across studies and facilitate the integration of findings. It is advisable to strengthen multicenter collaboration, increase sample sizes, and conduct long-term follow-up studies to assess the enduring effects of CCT and its applicability at various stages. Given the substantial variability in intervention duration and CCT systems among the included trials, minor discrepancies in results may be anticipated. It has been suggested that such research should be expanded to evaluate effectiveness in real-world applications, with a focus on implementation strategies, collaboration with health care systems, and treatment settings that optimize health care expenditures and maximize patient benefits [18,25,89]. Future studies should consider conducting subgroup analyses based on the type of CCT system used, and the severity of PSCI, contingent upon a sufficient number of experiments. Concurrently, there should be an increased focus on developing personalized treatment and intervention strategies. These strategies should be integrated with other rehabilitation measures and tailored precisely to meet the specific needs of individual patients to optimize intervention outcomes. In addition, the inherent complexity of CCT may present challenges for rehabilitation medical personnel and hinder the use of patients. To address these issues and facilitate the sustainable use of computerized rehabilitation, Nahas et al [90] have suggested the development of standardized guidelines for medical staff, along with the implementation of CCT acceptability and acceptance models to enhance patient engagement. Furthermore, larger, adequately powered trials are necessary to determine which specific CCT regimens are most effective in improving cognitive function, specific cognitive domains, and daily functioning in patients with PSCI over the long term. Although the English literature covers studies in multiple countries, it may also lead to studies of specific populations, regions, or cultural backgrounds being overlooked. In future studies, we plan to further address this issue by collaborating with researchers proficient in multiple languages or by incorporating other non–English language databases.

### Conclusions

In summary, based on moderate to high levels of evidence, we conclude that CCT is effective in improving general cognitive function, as well as attention and executive function, and quality of life in patients with PSCI. In addition, short-term high-frequency training was more effective than long-term low-frequency training. This provides important guidance for clinicians when designing interventions for CCT. Therefore, training tasks can be designed to target specific cognitive deficits such as memory, attention, or executive dysfunction. At the same time, health care providers should consider scheduling training as intensive sessions daily or multiple times per week to achieve more significant short-term improvements. After the initial effect of high-frequency training, a maintenance training program should be gradually designed to help patients consolidate the acquired cognitive function and prevent the decline of the training effect.

## Data Availability

All data generated or analyzed during this study were included in published articles. All data have been published in open-access journals.

## Appendix

Multimedia Appendix 1 PRISMA (Preferred Reporting Items for Systematic Reviews and Meta-Analyses) checklist.

Multimedia Appendix 2 Example search strategy.

Multimedia Appendix 3 Outcome indicators.

Multimedia Appendix 4 Characteristics of Included Studies.

Multimedia Appendix 5 The GRADE Summary of Findings for the Outcomes.

Multimedia Appendix 6 Sensitivity analysis results.

Multimedia Appendix 7 Forest plot of long-term analysis.

Multimedia Appendix 8 Results of other subgroup analysis of general cognitive function.

Multimedia Appendix 9 Funnel plot of publication bias.

## Abbreviations

CCT: computerized cognitive training
GRADE: Grading of Recommendations Assessment, Development, and Evaluation
LOTCA: Loewenstein Occupational Therapy Cognitive Assessment
MeSH: Medical Subject Headings
PICOS: Population, Intervention, Comparison, and Outcome
PRISMA: Preferred Reporting Items for Systematic Reviews and Meta-Analyses
PSCI: poststroke cognitive impairment
RCT: randomized controlled trial
SMD: standardized mean difference
