# Supplementary material for: The Effectiveness of Computerized Cognitive Training in Patients With Poststroke Cognitive Impairment: Systematic Review and Meta-Analysis
Source: J Med Internet Res. 2025 Jun 12;27:e73140. doi: 10.2196/73140 (PMC12203030; doi:10.2196/73140)
Supplement: Multimedia Appendix 2 [file jmir_v27i1e73140_app2.docx]

**Multimedia Appendix 2. Search strategy.**

Databases: PubMed, Web of Science, EMBASE, Cochrane Library, and Scopus. The laster update for the search is June 12, 2024 .

**PubMed：**

((((((((""Stroke""[Mesh]) OR ""Brain Infarction""[Mesh]) OR ""Cerebral Hemorrhage""[Mesh]) OR ""Cerebrovascular Disorders""[Mesh]) OR ""Brain Ischemia""[Mesh]) OR ((((((((((((((((((((((((((((((Strokes[Title/Abstract]) OR (Cerebrovascular Accident[Title/Abstract])) OR (Cerebrovascular Accidents[Title/Abstract])) OR (Cerebral Stroke[Title/Abstract])) OR (Cerebral Strokes[Title/Abstract])) OR (Stroke, Cerebral[Title/Abstract])) OR (Strokes, Cerebral[Title/Abstract])) OR (Cerebrovascular Apoplexy[Title/Abstract])) OR (Apoplexy, Cerebrovascular[Title/Abstract])) OR (Vascular Accident, Brain[Title/Abstract])) OR (Brain Vascular Accident[Title/Abstract])) OR (Brain Vascular Accidents[Title/Abstract])) OR (Vascular Accidents, Brain[Title/Abstract])) OR (Cerebrovascular Stroke[Title/Abstract])) OR (Cerebrovascular Strokes[Title/Abstract])) OR (Stroke, Cerebrovascular[Title/Abstract])) OR (Strokes, Cerebrovascular[Title/Abstract])) OR (Apoplexy[Title/Abstract])) OR (CVA[Title/Abstract])) OR (Cerebrovascular Accident[Title/Abstract])) OR (CVAs[Title/Abstract])) OR (Cerebrovascular Accident[Title/Abstract])) OR (Stroke, Acute[Title/Abstract])) OR (Acute Stroke[Title/Abstract])) OR (Acute Strokes[Title/Abstract])) OR (Strokes, Acute[Title/Abstract])) OR (Cerebrovascular Accident, Acute[Title/Abstract])) OR (Acute Cerebrovascular Accident[Title/Abstract])) OR (Acute Cerebrovascular Accidents[Title/Abstract])) OR (Cerebrovascular Accidents, Acute[Title/Abstract]))) OR ((((((((cerebrovascular disease[Title/Abstract]) OR (cerebrovascular diseases[Title/Abstract])) OR (Intracranial Hemorrhage[Title/Abstract])) OR (intracerebral hemorrhage[Title/Abstract])) OR (intracranial bleeding[Title/Abstract])) OR (ICH[Title/Abstract])) OR (poststroke*[Title/Abstract])) OR (post-stroke*[Title/Abstract]))) AND (((((""Cognitive Dysfunction""[Mesh]) OR ""Cognition Disorders""[Mesh]) OR ""Cognition""[Mesh]) OR ""Neurocognitive Disorders""[Mesh]) OR ((((((((((((((((((((((((((((Cognition Disorder[Title/Abstract]) OR (Cognition Defects[Title/Abstract])) OR (Cognition Defect[Title/Abstract])) OR (cognitive defect[Title/Abstract])) OR (cognitive defects[Title/Abstract])) OR (cognitive impairments[Title/Abstract])) OR (cognitive decline[Title/Abstract])) OR (cognitive status[Title/Abstract])) OR (cognitive function[Title/Abstract])) OR (cognitive functioning[Title/Abstract])) OR (cognitive dysfunctioning[Title/Abstract])) OR (MCI[Title/Abstract])) OR (Cogni*[Title/Abstract])) OR (Neurocogni*[Title/Abstract])) OR (cogniti* disorder*[Title/Abstract])) OR (cogniti* disruption*[Title/Abstract])) OR (cogniti* impair*[Title/Abstract])) OR (cogniti* abilil*[Title/Abstract])) OR (cognition[Title/Abstract])) OR (executive function[Title/Abstract])) OR (memory[Title/Abstract])) OR (problem solving[Title/Abstract])) OR (verbal learning[Title/Abstract])) OR (perception[Title/Abstract])) OR (visual perception[Title/Abstract])) OR (decision making[Title/Abstract])) OR (judgment[Title/Abstract])) OR (Attention[Title/Abstract])))) AND ((((((((((((((((((((((Computer aided cognitive training[Title/Abstract]) OR (Computer-assisted cognitive rehabilitation[Title/Abstract])) OR (computer assisted training[Title/Abstract])) OR (computer aided training[Title/Abstract])) OR (computerized cognitive training[Title/Abstract])) OR (computerized training[Title/Abstract])) OR (computional training[Title/Abstract])) OR (Software Training[Title/Abstract])) OR (Cognitive Training[Title/Abstract])) OR (rehabilitation training[Title/Abstract])) OR (Cognitive intervention[Title/Abstract])) OR (Computer Calculators[Title/Abstract])) OR (Programmable Calculators Computers[Title/Abstract])) OR (Digital Computers[Title/Abstract])) OR (Digital Computers Hardware[Title/Abstract])) OR (CACR[Title/Abstract])) OR (CACT[Title/Abstract])) OR (CCT[Title/Abstract])) OR (CCR[Title/Abstract])) OR (computer assisted therap*[Title/Abstract])) OR (computer-assisted therap*[Title/Abstract])) OR ((((((((((((((((((((((digital intervention[Title/Abstract]) OR (digital interventions[Title/Abstract])) OR (video game[Title/Abstract])) OR (video games[Title/Abstract])) OR (gamif*[Title/Abstract])) OR (digital health intervention[Title/Abstract])) OR (digital health interventions[Title/Abstract])) OR (e-health[Title/Abstract])) OR (m-health[Title/Abstract])) OR (computer game*[Title/Abstract])) OR (robot*[Title/Abstract])) OR (tablet*[Title/Abstract])) OR (phone*[Title/Abstract])) OR (ipad*[Title/Abstract])) OR (laptop*[Title/Abstract])) OR (virtual reality*[Title/Abstract])) OR (augmented reality*[Title/Abstract])) OR (artificial intelligence*[Title/Abstract])) OR (video*[Title/Abstract])) OR (comput*[Title/Abstract])) OR (software*[Title/Abstract])) OR (technology[Title/Abstract]))) Filters: from 2010 - 2024

**Web of Science:**

#1:TS=(Stroke OR Strokes OR “Cerebrovascular Accident” OR “Cerebrovascular Accidents” OR “Cerebral Stroke” OR “Cerebral Strokes” OR “Stroke, Cerebral” OR “Strokes, Cerebral” OR “Cerebrovascular Apoplexy” OR “Apoplexy, Cerebrovascular” OR “Vascular Accident, Brain” OR “Brain Vascular Accident” OR “Brain Vascular Accidents” OR “Vascular Accidents, Brain” OR “Cerebrovascular Stroke” OR “Cerebrovascular Strokes” OR “Stroke, Cerebrovascular” OR “Strokes, Cerebrovascular” OR “Apoplexy” OR CVA OR “Cerebrovascular Accident” OR “CVAs” OR “Cerebrovascular Accident” OR “Stroke, Acute” OR “Acute Stroke” OR “Acute Strokes” OR “Strokes, Acute” OR “Cerebrovascular Accident, Acute” OR “Acute Cerebrovascular Accident” OR “Acute Cerebrovascular Accidents” OR “Cerebrovascular Accidents, Acute” OR “Brain Infarction” OR “Cerebral Hemorrhage” OR “Cerebrovascular Disorders” OR “Brain Ischemia” OR “cerebrovascular disease” OR “cerebrovascular diseases” OR “Intracranial Hemorrhage” OR “intracerebral hemorrhage” OR “intracranial bleeding” OR “ICH” OR “poststroke*” OR “post-stroke*”) and Preprint Citation Index (Exclude – Database)

#2:TS=(“Cognitive Dysfunction” OR “Cognition Disorders” OR “Cognition” OR “Neurocognitive Disorders” OR “Cognition Disorder” OR “Cognition Defects” OR “Cognition Defect” OR “cognitive defect” OR “cognitive defects” OR “cognitive impairments” OR “cognitive decline” OR “cognitive status” OR “cognitive function” OR “cognitive functioning” OR “cognitive dysfunctioning” OR MCI OR “Cogni*” OR “Neurocogni*” OR “cogniti* disorder*” OR “cogniti* disruption*” OR “cogniti* impair*” OR “cogniti* abilil*” OR “cognition” OR “executive function” OR memory OR “problem solving” OR “verbal learning” OR perception OR “visual perception” OR “decision making” OR judgment OR Attention) and Preprint Citation Index (Exclude – Database)

#3:TS=(“Computer aided cognitive training” OR “Computer-assisted cognitive rehabilitation” OR “computer assisted training” OR “computer aided training” OR “computerized cognitive training” OR “computerized training” OR “computional training” OR “Software Training” OR “Cognitive Training” OR “rehabilitation training” OR “Cognitive intervention” OR “Computer Calculators” OR “Programmable Calculators Computers” OR “Digital Computers” OR “Digital Computers Hardware” OR CACR OR CACT OR CCT OR CCR OR “computer assisted therap*” OR “computer-assisted therap*” OR “digital intervention” OR “digital interventions” OR “video game” OR “video games” OR gamif* OR “digital health intervention” OR “digital health interventions” OR e-health OR m-health OR “computer game*” OR robot* OR tablet* OR phone* OR ipad* OR laptop* OR “virtual reality*” OR “augmented reality*” OR “artificial intelligence*” OR video* OR comput* OR software* OR technology) and Preprint Citation Index (Exclude – Database)

#4:#1 AND #2 AND #3 AND TS=(RCT* OR random* OR trial* OR experiment*) and Preprint Citation Index (Exclude – Database)

**EMBASE:**

#1:'cerebrovascular accident'/exp OR 'cerebrovascular accident' OR 'brain infarction'/exp OR 'brain infarction' OR 'brain hemorrhage'/exp OR 'brain hemorrhage' OR 'cerebrovascular disease'/exp OR 'cerebrovascular disease' OR 'brain ischemia'/exp OR 'brain ischemia'

#2:strokes:ti,ab,kw OR 'cerebrovascular accidents':ti,ab,kw OR 'cerebral stroke':ti,ab,kw OR 'cerebral strokes':ti,ab,kw OR 'stroke, cerebral':ti,ab,kw OR 'strokes, cerebral':ti,ab,kw OR 'cerebrovascular apoplexy':ti,ab,kw OR 'apoplexy, cerebrovascular':ti,ab,kw OR 'vascular accident, brain':ti,ab,kw OR 'brain vascular accident':ti,ab,kw OR 'brain vascular accidents':ti,ab,kw OR 'vascular accidents, brain':ti,ab,kw OR 'cerebrovascular stroke':ti,ab,kw OR 'cerebrovascular strokes':ti,ab,kw OR 'stroke, cerebrovascular':ti,ab,kw OR 'strokes, cerebrovascular':ti,ab,kw OR apoplexy:ti,ab,kw OR cva:ti,ab,kw OR cvas:ti,ab,kw OR 'cerebrovascular accident':ti,ab,kw OR 'stroke, acute':ti,ab,kw OR 'acute stroke':ti,ab,kw OR 'acute strokes':ti,ab,kw OR 'strokes, acute':ti,ab,kw OR 'cerebrovascular accident, acute':ti,ab,kw OR 'acute cerebrovascular accident':ti,ab,kw OR 'acute cerebrovascular accidents':ti,ab,kw OR 'cerebrovascular accidents, acute':ti,ab,kw OR 'cerebrovascular disease':ti,ab,kw OR 'cerebrovascular diseases':ti,ab,kw OR 'intracranial hemorrhage':ti,ab,kw OR 'intracerebral hemorrhage':ti,ab,kw OR 'intracranial bleeding':ti,ab,kw OR ich:ti,ab,kw OR poststroke*:ti,ab,kw OR 'post stroke*':ti,ab,kw

#3:#1 OR #2

#4:'cognitive defect'/exp OR 'cognition'/exp OR 'cognition disorder':ti,ab,kw OR 'cognition defects':ti,ab,kw OR 'cognition defect':ti,ab,kw OR 'cognitive defect':ti,ab,kw OR 'cognitive defects':ti,ab,kw OR 'cognitive impairments':ti,ab,kw OR 'cognitive decline':ti,ab,kw OR 'cognitive status':ti,ab,kw OR 'cognitive function':ti,ab,kw OR 'cognitive functioning':ti,ab,kw OR 'cognitive dysfunctioning':ti,ab,kw OR mci:ti,ab,kw OR cogni*:ti,ab,kw OR neurocogni*:ti,ab,kw OR 'cogniti* disorder*':ti,ab,kw OR 'cogniti* disruption*':ti,ab,kw OR 'cogniti* impair*':ti,ab,kw OR 'cogniti* abilil*':ti,ab,kw OR cognition:ti,ab,kw OR 'executive function':ti,ab,kw OR memory:ti,ab,kw OR 'problem solving':ti,ab,kw OR 'verbal learning':ti,ab,kw OR perception:ti,ab,kw OR 'visual perception':ti,ab,kw OR 'decision making':ti,ab,kw OR judgment:ti,ab,kw OR attention:ti,ab,kw

#5:'computer aided cognitive training':ti,ab,kw OR 'computer-assisted cognitive rehabilitation':ti,ab,kw OR 'computer assisted training':ti,ab,kw OR 'computer aided training':ti,ab,kw OR 'computerized cognitive training':ti,ab,kw OR 'computerized training':ti,ab,kw OR 'computional training':ti,ab,kw OR 'software training':ti,ab,kw OR 'cognitive training':ti,ab,kw OR 'rehabilitation training':ti,ab,kw OR 'cognitive intervention':ti,ab,kw OR 'computer calculators':ti,ab,kw OR 'programmable calculators computers':ti,ab,kw OR 'digital computers':ti,ab,kw OR 'digital computers hardware':ti,ab,kw OR cacr:ti,ab,kw OR cact:ti,ab,kw OR cct:ti,ab,kw OR ccr:ti,ab,kw OR 'computer assisted therap*':ti,ab,kw OR 'computer-assisted therap*':ti,ab,kw OR 'digital intervention':ti,ab,kw OR 'digital interventions':ti,ab,kw OR 'video game':ti,ab,kw OR 'video games':ti,ab,kw OR gamif*:ti,ab,kw OR 'digital health intervention':ti,ab,kw OR 'digital health interventions':ti,ab,kw OR 'e health':ti,ab,kw OR 'm health':ti,ab,kw OR 'computer game*':ti,ab,kw OR robot*:ti,ab,kw OR tablet*:ti,ab,kw OR phone*:ti,ab,kw OR ipad*:ti,ab,kw OR laptop*:ti,ab,kw OR 'virtual reality*':ti,ab,kw OR 'augmented reality*':ti,ab,kw OR 'artificial intelligence*':ti,ab,kw OR video*:ti,ab,kw OR comput*:ti,ab,kw OR software*:ti,ab,kw OR technology:ti,ab,kw

#6: rct* OR random* OR trial* OR experiment*

#7:#3 AND #4 AND #5 AND #6

#7 AND (2010:py OR 2011:py OR 2012:py OR 2013:py OR 2014:py OR 2015:py OR 2016:py OR 2017:py OR 2018:py OR 2019:py OR 2020:py OR 2021:py OR 2022:py OR 2023:py OR 2024:py)

**Cochrane Library:**

#1:MeSH descriptor: [Stroke] explode all trees

#2:MeSH descriptor: [Brain Infarction] explode all trees

#3:MeSH descriptor: [Cerebral Hemorrhage] explode all trees

#4:MeSH descriptor: [Cerebrovascular Disorders] explode all trees

#5:MeSH descriptor: [Brain Ischemia] explode all trees

#6:(Strokes):ti,ab,kw OR ('Cerebrovascular Accident'):ti,ab,kw OR ('Cerebrovascular Accidents'):ti,ab,kw OR ('Cerebral Stroke'):ti,ab,kw OR ('Cerebral Strokes'):ti,ab,kw

#7:(Strokes or 'Cerebrovascular Accident' or 'Cerebrovascular Accidents' or 'Cerebral Stroke' or 'Cerebral Strokes' or 'Stroke, Cerebral' or 'Strokes, Cerebral' or 'Cerebrovascular Apoplexy' or 'Apoplexy, Cerebrovascular' or Vascular Accident, Brain' or 'Brain Vascular Accident' or 'Brain Vascular Accidents' or 'Vascular Accidents, Brain' or 'Cerebrovascular Stroke' or 'Cerebrovascular Strokes' or 'Stroke, Cerebrovascular' or 'Strokes, Cerebrovascular' or 'Apoplexy' or 'CVA ' or 'Cerebrovascular Accident' or 'CVAs ' or 'Stroke, Acute' or 'Acute Stroke' or 'Acute Strokes' or 'Strokes, Acute' or 'Cerebrovascular Accident, Acute' or 'Acute Cerebrovascular Accident' or 'Acute Cerebrovascular Accidents' or 'Cerebrovascular Accidents, Acute' orcerebrovascular disease' or 'cerebrovascular diseases' or 'Intracranial Hemorrhage' or 'intracerebral hemorrhage' or 'intracranial bleeding' or 'ICH' or 'poststroke*' or 'post-stroke*'):ti,ab,kw

#8:#1 or #2 or #3 or #4 or #5 or #6 or #7

#9:MeSH descriptor: [Cognitive Dysfunction] explode all trees

#10:MeSH descriptor: [Cognition Disorders] explode all trees

#11:MeSH descriptor: [Cognition] explode all trees

#12:MeSH descriptor: [Neurocognitive Disorders] explode all trees

#13:('Cognition Disorder' or 'Cognition Defects' or 'Cognition Defect' or 'cognitive defect' or 'cognitive defects' or 'cognitive impairments' or 'cognitive decline' or 'cognitive status' or 'cognitive function' or 'cognitive functioning' or 'cognitive dysfunctioning' or 'MCI' or 'Cogni*' or 'Neurocogni*' or 'cogniti* disorder*' or 'cogniti* disruption*' or 'cogniti* impair*' or 'cogniti* abilil*' or 'cognition' or 'executive function' or 'memory' or 'problem solving' or 'verbal learning' or 'perception' or 'visual perception' or 'decision making' or 'judgment' or 'Attention'):ti,ab,kw

#14:#9 or #10 or #11 or #12 or #13

#15:('Computer aided cognitive training' or 'Computer-assisted cognitive rehabilitation' or 'computer assisted training' or 'computer aided training' or 'computerized cognitive training' or 'computerized training' or 'computional training' or 'Software Training' or 'Cognitive Training' or 'rehabilitation training' or 'Cognitive intervention' or 'Computer Calculators' or 'Programmable Calculators Computers' or 'Digital Computers' or 'Digital Computers Hardware' or 'CACR' or 'CACT' or 'CCT' or 'CCR' or 'computer assisted therap*' or 'computer-assisted therap*'or 'digital intervention' or 'digital interventions' or 'video game' or 'video games' or 'gamif*' or 'digital health intervention' or 'digital health interventions' or 'e-health' or 'm-health' or 'computer game*' or robot* or tablet* or phone* or ipad* or laptop* or 'virtual reality*' or 'augmented reality*' or 'artificial intelligence*' or video* or comput* or software* or technology):ti,ab,kw

#16:#8 and #14 and #15

**Scopus:**

( TITLE-ABS-KEY ( stroke OR strokes OR "cerebrovascular accident" OR "cerebrovascular accidents" OR "cerebral stroke" OR "cerebral strokes" OR "stroke, cerebral" OR "strokes, cerebral" OR "cerebrovascular apoplexy" OR "apoplexy, cerebrovascular" OR "vascular accident, brain" OR "brain vascular accident" OR "brain vascular accidents" OR "vascular accidents, brain" OR "cerebrovascular stroke" OR "cerebrovascular strokes" OR "stroke, cerebrovascular" OR "strokes, cerebrovascular" OR "apoplexy" OR cva OR "cerebrovascular accident" OR "cvas" OR "cerebrovascular accident" OR "stroke, acute" OR "acute stroke" OR "acute strokes" OR "strokes, acute" OR "cerebrovascular accident, acute" OR "acute cerebrovascular accident" OR "acute cerebrovascular accidents" OR "cerebrovascular accidents, acute" OR "brain infarction" OR "cerebral hemorrhage" OR "cerebrovascular disorders" OR "brain ischemia" OR "cerebrovascular disease" OR "cerebrovascular diseases" OR "intracranial hemorrhage" OR "intracerebral hemorrhage" OR "intracranial bleeding" OR "ich" OR "poststroke*" OR "post-stroke*" ) AND TITLE-ABS-KEY ( "cognitive dysfunction" OR "cognition disorders" OR "cognition" OR "neurocognitive disorders" OR "cognition disorder" OR "cognition defects" OR "cognition defect" OR "cognitive defect" OR "cognitive defects" OR "cognitive impairments" OR "cognitive decline" OR "cognitive status" OR "cognitive function" OR "cognitive functioning" OR "cognitive dysfunctioning" OR mci OR "cogni*" OR "neurocogni*" OR "cogniti* disorder*" OR "cogniti* disruption*" OR "cogniti* impair*" OR "cogniti* abilil*" OR "cognition" OR "executive function" OR memory OR "problem solving" OR "verbal learning" OR perception OR "visual perception" OR "decision making" OR judgment OR attention ) AND TITLE-ABS-KEY ( "computer aided cognitive training" OR "computer-assisted cognitive rehabilitation" OR "computer assisted training" OR "computer aided training" OR "computerized cognitive training" OR "computerized training" OR "computional training" OR "software training" OR "cognitive training" OR "rehabilitation training" OR "cognitive intervention" OR "computer calculators" OR "programmable calculators computers" OR "digital computers" OR "digital computers hardware" OR cacr OR cact OR cct OR ccr OR "computer assisted therap*" OR "computer-assisted therap*" OR "digital intervention" OR "digital interventions" OR "video game" OR "video games" OR gamif* OR "digital health intervention" OR "digital health interventions" OR e-health OR m-health OR "computer game*" OR robot* OR tablet* OR phone* OR ipad* OR laptop* OR "virtual reality*" OR "augmented reality*" OR "artificial intelligence*" OR video* OR comput* OR software* OR technology ) AND TITLE-ABS-KEY ( rct* OR random* OR trial* OR experiment* ) )
