# Supplementary material for: The Effectiveness of Computerized Cognitive Training in Patients With Poststroke Cognitive Impairment: Systematic Review and Meta-Analysis
Source: J Med Internet Res. 2025 Jun 12;27:e73140. doi: 10.2196/73140 (PMC12203030; doi:10.2196/73140)
Supplement: Multimedia Appendix 3 [file jmir_v27i1e73140_app3.docx]

**Multimedia Appendix 3. Outcome indicators.**

**General cognitive:**

1. Montreal Cognitive Assessment Scale (MoCA)

2. Loewenstein occupational therapy cognitive assessment (LOCTA)

3. Minimum Mental State Examination (MMSE)

4. the Cognitive Failure Questionnaire (CFQ)

**Attention:**

1. Averaged Standardized Composite Scores (ASCS)

2. Trail Making Test-A (TMT-A). This scale is based on response time, i.e., fewer scores mean better functioning, so a negative effect size means improved functioning in the test group compared to the control group.

3. Addenbrooke Cognitive Examination (ACE)

4. Rivermead Behavioral Memory Test-Third Edition—Global Memory Index (RBMT- GMI)

5. the Attentive Matrices (AM)

6. Shulte's table

**Memory:**

1. ACE-memory

2. Digital span test (DST)

3. Goal Attainment Scaling (GAS)

**Executive functions:**

1. Delis-Kaplan Executive Function System (D-Kefs)

2. Digit Symbol Coding (DSC)

3. clock drawing test (CDT)

**Language:**

1. Wechsler Adult Intelligence Scale III (WAIS)

2. ACE-language

3. Semantic Verbal Fluency (SVF)

**Quality of life:**

1. Functional Independence Measure (FIM)

2. Modified Barthel Index (MBI)

3. Stroke Impact Scale (SIS) 3.0

4. Instrumental activities of daily living (IADL)

**Motor function:**

1. Fugl-Meyer Assessment for the Upper Limb (FM-UE)

2. Simplified Fugl-Meyer Assessment (FMA)
