# Supplementary material for: The Effectiveness of Computerized Cognitive Training in Patients With Poststroke Cognitive Impairment: Systematic Review and Meta-Analysis
Source: J Med Internet Res. 2025 Jun 12;27:e73140. doi: 10.2196/73140 (PMC12203030; doi:10.2196/73140)
Supplement: Multimedia Appendix 4 [file jmir_v27i1e73140_app4.docx]

**Multimedia Appendix 4. Characteristics of Included Studies**

| **Author, year, country** | **Intervention Group** | **Intervention** | **Control Group** | **Comparison** | **computerized training tools** | **Frequency Duration of Session** | **Mode of supervision** | **Follow up** | **Cognitive domains targeted by CCT** | **Outcome measures** |
| --- | --- | --- | --- | --- | --- | --- | --- | --- | --- | --- |
| Baltaduonienė D, 2019, Lithuania | T2 group (n=41) | OT+ computerized cognitive training programme | T1 group (n=40) | Individual occupational therapy (OT) sessions, with the conventional “pencil–and–paper” cognition training tasks. | PssCogRehab 2012, USA | 45 min, 5 sessions per week, for 6 weeks | Supervised in the hospital | \ | Memory, problem solving, attention, and spatial perception | General cognitive: MoCa |
| Faria AL, 2016, Portugal | experimental (n=9) | VR- based intervention (Simulation of ADL’s with the Reh@City) | control (n=9) | conventional rehabilitation | the Reh@City | 20 min, 12 sessions, distributed from 4 to 6 weeks | Supervised in the hospital | \ | Attention, memory, executive function | General cognitive: MMSE  Attention: TMT-A  Memory: ACE-memory  Executive function: WAIS-Ⅲ Picture Arrangement  Language: ACE-language  Quality of life: SIS-ADL |
| Faria AL, 2020, Portugal | VR-based intervention: (n=17) | adaptive cognitive training through everyday tasks VR simulations | Task Generator (n=19) | TG: content equivalent and adaptive paper-and-pencil training | [Reh@City v2.0](mailto:Reh@City%20v2.0) | 12 sessions, for 4 weeks | Supervised in the hospital | 1 month | Memory, attention, executive function, language | General cognitive: MoCa  Attention: TMT-A  Memory: WAIS-Ⅲ Digit Span  Executive function: WAIS-Ⅲ DSC codification  Language: WAIS-Ⅲ vocabulary |
| Youze H, 2021, China | Computer‑aided self‑regulation learning (CA‑SRL) group(n=23) | CA-SRL training, CACT and basic ADL training. | Traditional learning (TL) group(n=25) | traditional cognitive training with the same training dose rather than CACT | CACT developed by the cognitive rehabilitation research team of Hong Kong Polytechnic University, Hong Kong University and Fujian University of Traditional Chinese Medicine | 30 min, 5 sessions per week, for 3 weeks | Supervised in the hospital | \ | Visual perception, visual attention, sustained attention, working memory | General cognitive: MoCa  Quality of life: MBI  Motor function: FMA |
| Ho HY, 2022, Taiwan | intervention group (n = 19) | receiving CCT with Lumosity software | the control group (n = 20) | receiving conventional cognitive training | Lumosity software. (www.lumosity.com) | 20 min, 2 sessions per week, for 12 weeks | Supervised in the hospital | 1 month | Information processing speed, attention, and memory | General cognitive: MoCa  Memory: WMS-Ⅲ Digit Span  Quality of life: SIS-ADL |
| Jiang C, 2016, China | RehaCom training group (n=51) | conventional therapy＋ CACR training using RehaCom software translated into Chinese. | control group (n=49) | received only traditional rehabilitation therapy, including basic treatment and health education. | RehaCom, Hasomed Inc., Germany (http://www.hasomed.de) | 30 min, 5 sessions per week, for 12 weeks | Supervised in the hospital | \ | Attention, memory, executive function, visual field | General cognitive: MoCa  Quality of life: FIM |
| Jonsdottir J, 2021, Italy | home rehabilitation (HH, n = 11) | telerehabilitation at home (HomeHEAD) carried out with Kinect | follow usual care (UC, n = 23) | Treatment as Usual at home | Kinect (Microsoft, WA, USA) and Leap Motion (Leap Motion Inc., CA, USA) | 45 min, 5 sessions per week, for 12 weeks | Supervised at home | 3 months | Attention, memory, executive function and occupational activities | General cognitive: MoCa  Attention: RBMT-GMI |
| Luca RD, 2018, Italy | the experimental group (EG: n = 20) | the traditional cognitive rehabilitation + pc-based Erica training | the control group (CG: n = 15) | the traditional cognitive rehabilitation | Italian computerized cognitive tool, Erica (www.erica.giunti.it) | 45 min, 3 sessions per week, for 8 weeks | Supervised in the hospital | \ | Attention, memory, spatial cognition, verbal and nonverbal executive functions | General cognitive: MMSE  Attention: the Attentive Matrices (AM)  Memory: Digit Span  Language: Verbal Fluency |
| Maier M, 2020, Spain | experimental group (EG, n=16) | provided through the RGS set-up, a VR-based rehabilitation tool. | control group (CG, n=14) | individual cognitive tasks that had to be completed at home | the RGS | 30 min, 5 sessions per week, for 6 weeks | Supervised in the hospital | 3 months | Attention, memory, executive function | General cognitive: MoCa  Attention: ASCS  Memory: WAIS Digit Span  Executive function: WAIS DSC  Quality of life: BI  Motor function: FMA-UE |
| Park JH, 2015, Korea | experimental group (EG, n=15) | CBCR with the CoTras program | control group (CG, n=15) | conventional cognitive rehabilitation | Korean computer-based cognitive rehabilitation program (CBCR) (Netblue Co., Ltd, Korea) | 30 min, 5 sessions per week, for 4 weeks | Supervised in the hospital | \ | Visual perception, attention, memory, orientation | General cognitive: LOTCA |
| Poulin V, 2017, Canada | COMPUTER training (n=5) | Computerized executive function training, Attentional software | CO-OP (n=6) | Cognitive Orientation to daily Occupational Performance | NeuroActiveⓇ software (Brain Centre International Inc., Quebec, QC), Attentional software (Le Re´seau Psychotech Inc., Quebec, QC) | 60 min, 2 sessions per week, for 8 weeks | Supervised at home | 1 month | Attention, working memory | Attention: TMT-A  Memory: WAIS-Ⅳ Digit Span  Executive function: D-KEFS CWIT |
| Prokopenko SV, 2013, Russia | intervention group (n=24) | computer programs + standard treatment | control group (n=19) | standard treatment | computerized Schulte's tables and the computer-based “figure-background” test | 30 min per day, for 2 weeks | Supervised in the hospital | \ | Attention, visual and spatial gnosis | General cognitive: MoCa  Attention: Shulte's table  Executive function: CDT  Quality of life: IADL |
| Ressner P, 2018, Czech Republic | treatment group (n=19) | computer-based cognitive rehabilitation + traditional rehabilitation | control group (n=14) | traditional rehabilitation | CBCR using commercially available software | 60 min, 2 sessions per week, for 12 weeks | Supervised in the hospital | \ | Attention, memory, visuospatial skills, and executive functions | General cognitive: MMSE  Attention: ACE-R attention and concentration  Memory: ACE-R memory  Language: ACE-R language |
| van de Ven RM, Buitenweg JI.V, 2017, The Netherlands | intervention group (n = 38) | computer-based cognitive flexibility training | active control group (i.e., mock training; n = 35) | Cognitive flexibility training, Mock training. | the preexisting brain training website (www.braingymmer.com) | 30 min, 5 sessions per week, for 12 weeks | Supervised at home | \ | Working memory, attention, reasoning | Attention: TMT-A  Memory: ACE-memory  Executive function: DKEF TMT |
| van de Ven RM, Murre JM. J, 2017, The Netherlands | intervention group (n = 38) | carried out by means of a professionally programmed website | an active control group (n = 35) | mock training | a professionally programmed website (www.braingymmer.com) | 30 min, 5 sessions per week, for 12weeks | Supervised at home | \ | Attention, memory, and reasoning | General cognitive: CFQ  Quality of life: IADL |
| Withiel TD, 2019, Australia | Computer training (n = 22) | computerized cognitive training (CCT) intervention | Wait control (n = 19) | offered a memory intervention | LumosityTM | 30 min, 5 sessions per week, for 6 weeks | Supervised at home | 1.5 months | Memory | Memory: GAS |
| Yao Z, 2023, China | Experimental group (n = 12) | routine occupational therapy training＋digital occupational training | Control group (n = 12) | routine occupational therapy (OT) for 1 h each day | Digital Occupational Training System | 60 min, 5 sessions per week, for 8 weeks | Supervised in the hospital | \ | Attention, memory, logical reasoning, calculation, hand-eye coordination | General cognitive: MoCa  Quality of life: MBI  Motor function: FMA-UE |
| ZC Lin, 2014, China | computer-assisted cognitive training (n=16) | computer-assisted cognitive training | no training (n=18) | no training | RehaCom software | 60 min, 6 sessions per week, for 10 weeks | Supervised in the hospital | \ | Memory, executive function | Attention: TMT-A  Memory: WMS Ⅱ MQ |
| Zucchella C, 2014, Italy | study group (SG, n=45) | therapist-guided computer exercises,  using two software programs | control group (CG, n=47) | a sham intervention | "Una palestra per la mente" and "Training di riabilitazione cognitiva" | 60 min, 4 sessions per week, for 4 weeks | Supervised in the hospital | \ | Attention, memory, executive function | General cognitive: MMSE  Attention: TMT-A  Memory: Digit Span  Language: Verbal Fluency  Quality of life: FIM |

Abbreviation:

OT=occupational therapy; VR= Virtual Reality; ADL= activities of daily living; TG=task generator; CCT= computerized cognitive training; CACT= computer aided cognitive training; CACR= computer aided cognitive rehabilitation; CBCR= computer-based cognitive rehabilitation; CCR= computerized cognitive rehabilitation; MoCa= Montreal Cognitive Assessment Scale; MMSE= Minimum Mental State Examination; LOTCA= Loewenstein occupational therapy cognitive assessment; CFQ= the Cognitive Failure Questionnaire; ASCS= Averaged Standardized Composite Scores; TMT-A= Trail Making Test-A; ACE= Addenbrooke Cognitive Examination; RBMT- GMI= Rivermead Behavioral Memory Test-Third Edition—Global Memory Index; AM= the Attentive Matrices; DST= Digital span test; GAS= Goal Attainment Scaling; WAIS= Wechsler Adult Intelligence Scale; FIM= Functional Independence Measure; MBI= Modified Barthel Index; SIS= Stroke Impact Scale; IADL= Instrumental activities of daily living; FM-UE= Fugl-Meyer Assessment for the Upper Limb; FMA= Simplified Fugl-Meyer Assessment.
