# Supplementary material for: The Effectiveness of Computerized Cognitive Training in Patients With Poststroke Cognitive Impairment: Systematic Review and Meta-Analysis
Source: J Med Internet Res. 2025 Jun 12;27:e73140. doi: 10.2196/73140 (PMC12203030; doi:10.2196/73140)
Supplement: Multimedia Appendix 5 [file jmir_v27i1e73140_app5.docx]

| **Multimedia Appendix 5. The GRADE Summary of Findings for the Outcomes** |
| --- |
| **CCT compared to usual care / routine rehabilitation training treatment for post-stroke cognitive impairment** |

**Patient or population:** post-stroke cognitive impairment

**Setting:** CCT compared with usual care / routine rehabilitation training treatment

**Intervention:** CCT

**Comparison:** Usual care / Routine rehabilitation training treatment

| **Certainty assessment** | | | | | | | **№ of patients** | | **Effect** | | **Certainty** | **Importance** |
| --- | --- | --- | --- | --- | --- | --- | --- | --- | --- | --- | --- | --- |
| **№ of studies** | **Study design** | **Risk of bias** | **Inconsistency** | **Indirectness** | **Imprecision** | **Other considerations** | **CCT** | **Usual care / Routine rehabilitation training treatment** | **Relative (95% CI)** | **Absolute (95% CI)** |  |  |
| **General cognitive** | | | | | | | | | | | | |
| 15 | randomised trials | not serious | serious^a^ | not serious | not serious | none | 360 | 356 | - | SMD **0.46 SD higher** (0.21 higher to 0.71 higher) | ⨁⨁⨁◯ Moderate^a,^ | CRITICAL |
| **Attention** | | | | | | | | | | | | |
| 11 | randomised trials | not serious | not serious | not serious | not serious | none | 219 | 218 | - | SMD **0.45 SD lower** (0.64 lower to 0.25 lower) | ⨁⨁⨁⨁ High | CRITICAL |
| **Memory** | | | | | | | | | | | | |
| 11 | randomised trials | not serious | serious^a^ | not serious | serious^c^ | publication bias strongly suspected^b^ | 225 | 215 | - | SMD **0.42 SD lower** (0.06 lower to 0.89 higher) | ⨁◯◯◯ Very low^a,b,c^ | CRITICAL |
| **Executive functions** | | | | | | | | | | | | |
| 6 | randomised trials | not serious | not serious | not serious | not serious | publication bias strongly suspected^b^ | 108 | 101 | - | SMD **0.39 SD higher** (0.12 higher to 0.67 higher) | ⨁⨁⨁◯ Moderate^b^ | CRITICAL |
| **Language** | | | | | | | | | | | | |
| 5 | randomised trials | not serious | not serious | not serious | serious^c^ | publication bias strongly suspected^b^ | 110 | 104 | - | SMD **0.21 SD higher** (0.06 lower to 0.48 higher) | ⨁⨁◯◯ Low^b,c^ | CRITICAL |
| **Quality of life** | | | | | | | | | | | | |
| 9 | randomised trials | not serious | not serious | not serious | not serious | none | 237 | 230 | - | SMD **0.34 SD higher** (0.15 higher to 0.53 higher) | ⨁⨁⨁⨁ High | IMPORTANT |
| **Motor function** | | | | | | | | | | | | |
| 3 | randomised trials | not serious | serious^a^ | not serious | serious^c^ | none | 51 | 51 | - | SMD **0.11 SD higher** (0.58 lower to 0.79 higher) | ⨁⨁◯◯ Low^a,c^ | NOT IMPORTANT |

**CI:** confidence interval; **SMD:** standardised mean difference

#### Explanations

a. heterogeneity in the I^2^ test >50%

b. 25% of participants were from trials at high overall risk of bias

c. the 95% CI crossed 0
