# Supplementary material for: The Effectiveness of Computerized Cognitive Training in Patients With Poststroke Cognitive Impairment: Systematic Review and Meta-Analysis
Source: J Med Internet Res. 2025 Jun 12;27:e73140. doi: 10.2196/73140 (PMC12203030; doi:10.2196/73140)
Supplement: Multimedia Appendix 6 [file jmir_v27i1e73140_app6.docx]

**Multimedia Appendix 6.1 Sensitivity analysis results for general cognitive after excluding Park JH, 2015.**

**
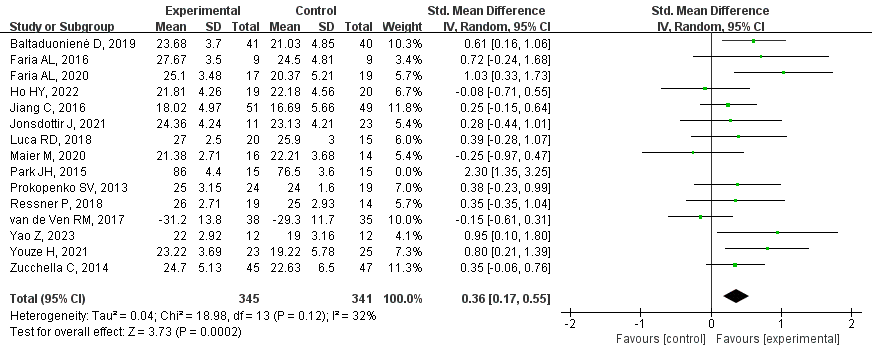
**

**Multimedia Appendix File 6.2 Sensitivity analysis results for memory after excluding Withiel TD, 2019.**

**
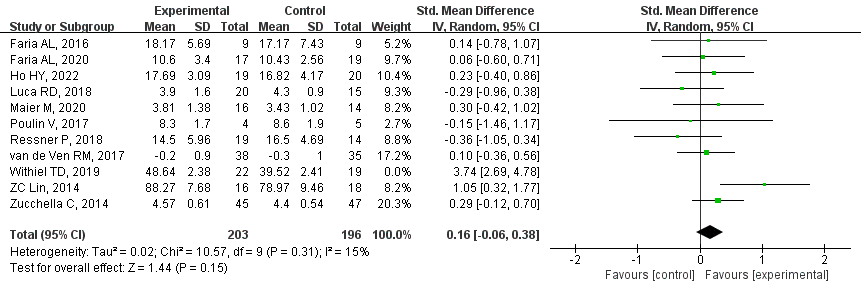
**
