# Supplementary material for: The Effectiveness of Computerized Cognitive Training in Patients With Poststroke Cognitive Impairment: Systematic Review and Meta-Analysis
Source: J Med Internet Res. 2025 Jun 12;27:e73140. doi: 10.2196/73140 (PMC12203030; doi:10.2196/73140)

**Multimedia Appendix 7.1 Forest plot of long-term analysis of general cognitive.**


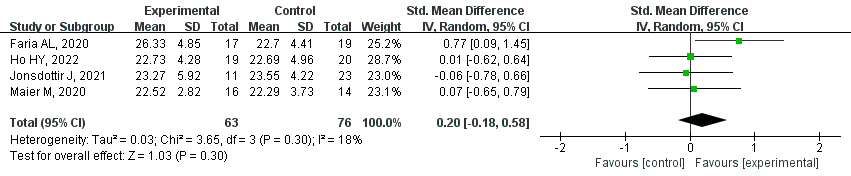


**Multimedia Appendix 7.2 Forest plot of long-term analysis of attention.**


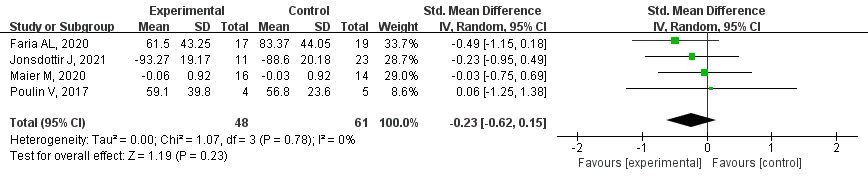


**Multimedia Appendix 7.3 Forest plot of long-term analysis of memory.**


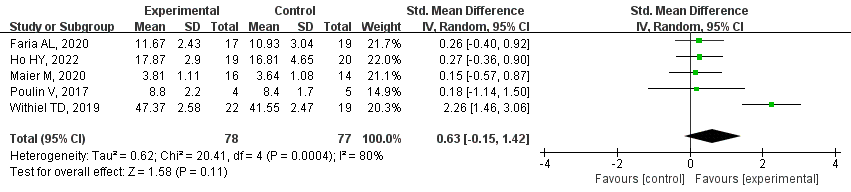


**Multimedia Appendix 7.4 Forest plot of long-term analysis of executive function.**


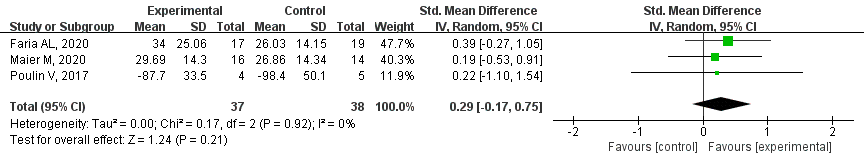

Supplement: Multimedia Appendix 7 [file jmir_v27i1e73140_app7.docx]
