# Supplementary material for: The Effectiveness of Computerized Cognitive Training in Patients With Poststroke Cognitive Impairment: Systematic Review and Meta-Analysis
Source: J Med Internet Res. 2025 Jun 12;27:e73140. doi: 10.2196/73140 (PMC12203030; doi:10.2196/73140)
Supplement: Multimedia Appendix 8 [file jmir_v27i1e73140_app8.docx]

**Multimedia Appendix 8**

**Results of other subgroup analysis of general cognitive function**

| **Groups** | **n** | **SMD** | **95%CI** | ***I²*（%）** | ***P*** |
| --- | --- | --- | --- | --- | --- |
| **Intervention frequency** |  |  |  |  | **.12** |
| High frequency/ Long duration  (>3d/w, >6 weeks) | 4 | 0.23 | (-0.14, 0.61) | 43.0 | .22 |
| Low frequency/ Long duration  (≤3d/w, >6 weeks) | 3 | 0.20 | (-0.18, 0.59) | 0.0 | .30 |
| High frequency/ Short duration  (>3d/w, ≤6 weeks) | 6 | 0.63 | (0.15, 1.10) | 75.0 | .01 |
| Low frequency/ Short duration  (≤3d/w, ≤6weeks) | 2 | 0.92 | (0.36, 1.49) | 0.0 | .001 |
| **Intervention in the control group** |  |  |  |  | **.56** |
| Active controls (traditional cognitive training) | 8 | 0.53 | (0.06, 0.99) | 78.0 | .03 |
| Passive controls or usual care | 7 | 0.37 | (0.16, 0.59) | 0.0 | <.001 |
| **Mode of supervision** |  |  |  |  | **.02** |
| Supervised in the hospital | 13 | 0.53 | (0.27, 0.80) | 58.0 | <.001 |
| Supervised at home | 2 | -0.02 | (-0.41, 0.37) | 0.0 | .91 |
