# Supplementary material for: The Effectiveness of Computerized Cognitive Training in Patients With Poststroke Cognitive Impairment: Systematic Review and Meta-Analysis
Source: J Med Internet Res. 2025 Jun 12;27:e73140. doi: 10.2196/73140 (PMC12203030; doi:10.2196/73140)

**Multimedia Appendix 9.1 Funnel plot of publication bias for General cognitive.**


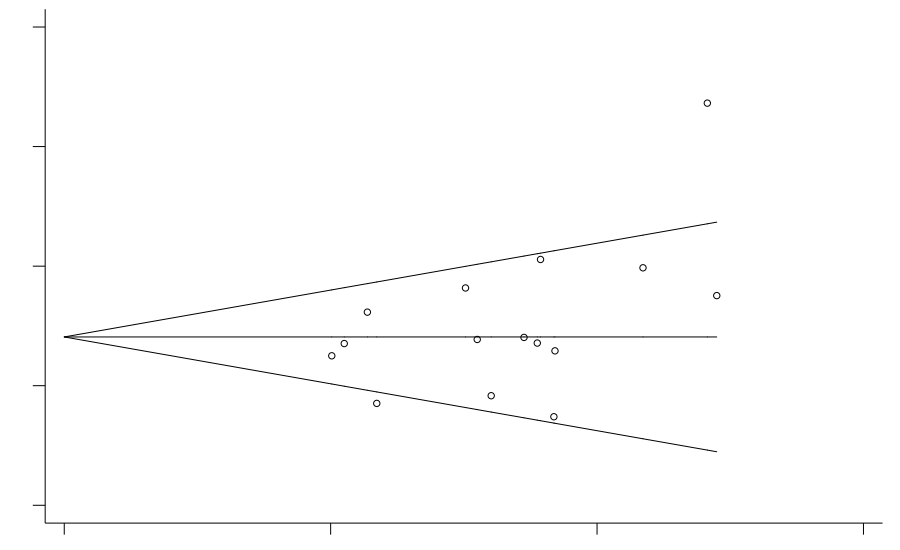


**Multimedia Appendix 9.2 Funnel plot of publication bias for Attention.**


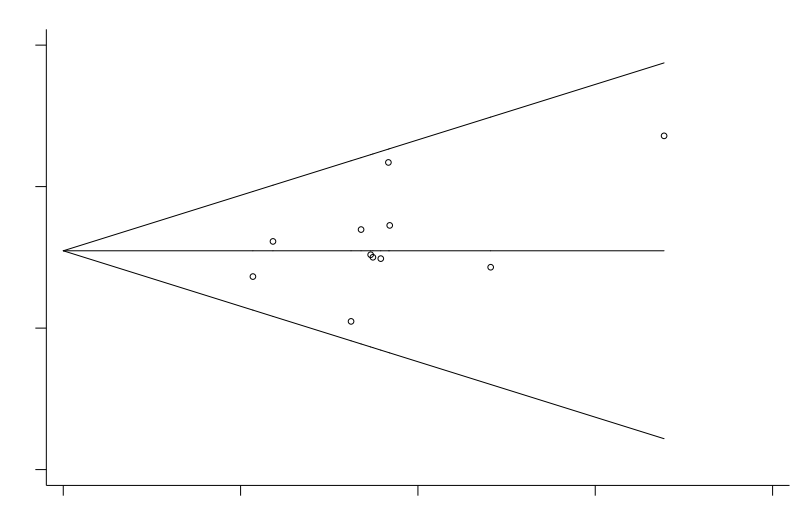


**Multimedia Appendix 9.3 Funnel plot of publication bias for Memory.**


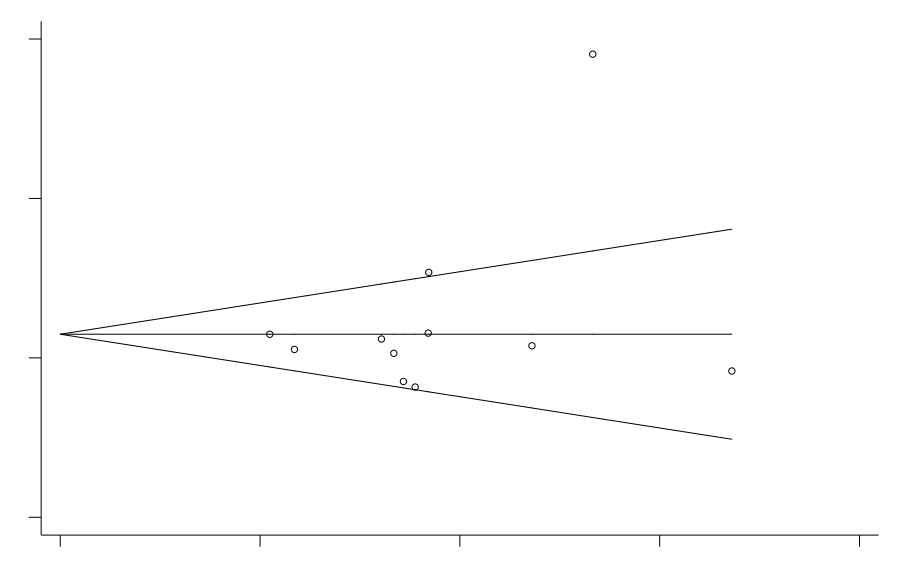

Supplement: Multimedia Appendix 9 [file jmir_v27i1e73140_app9.docx]
